# Acquisition of neural fate by combination of BMP blockade and chromatin modification

**DOI:** 10.1016/j.isci.2023.107887

**Published:** 2023-09-09

**Authors:** Agnes Lee Chen Ong, Toshiya Kokaji, Arisa Kishi, Yoshihiro Takihara, Takuma Shinozuka, Ren Shimamoto, Ayako Isotani, Manabu Shirai, Noriaki Sasai

**Affiliations:** 1 Division of Biological Sciences, Nara Institute of Science and Technology, 8916-5 Takayama-cho, Ikoma 630-0192, Japan; 2 Data-driven biology, NAIST Data Science Center, Nara Institute of Science and Technology, 8916-5 Takayama-cho, Ikoma 630-0192, Japan; 3 Research Institute for Radiation Biology and Medicine, Hiroshima University, 1-2-3, Kasumi, Minami-ku, Hiroshima 734-0037, Japan; 4 Omics Research Center (ORC), National Cerebral and Cardiovascular Center, 6-1 Kishibe Shinmachi, Suita, Osaka 564-8565, Japan

**Keywords:** Epigenetics, Molecular biology, Neuroscience, Stem cells research, Transcriptomics

## Abstract

Neural induction is a process where naive cells are converted into committed cells with neural characteristics, and it occurs at the earliest step during embryogenesis. Although the signaling molecules and chromatin remodeling for neural induction have been identified, the mutual relationships between these molecules are yet to be fully understood.

By taking advantage of the neural differentiation system of mouse embryonic stem (ES) cells, we discovered that the BMP signal regulates the expression of several polycomb repressor complex (PRC) component genes. We particularly focused on Polyhomeotic Homolog 1 (Phc1) and established *Phc1*-knockout (*Phc1*-KO) ES cells. We found that *Phc1*-KO failed to acquire the neural fate, and the cells remained in pluripotent or primitive non-neural states. Chromatin accessibility analysis suggests that Phc1 is essential for chromatin packing. Aberrant upregulation of the BMP signal was confirmed in the *Phc1* homozygotic mutant embryos. Taken together, Phc1 is required for neural differentiation through epigenetic modification.

## Highlights

• The Phc1 expression is regulated by BMP
• Phc1 is required to promote *in vitro* neural differentiation
• The chromatin loci at pluripotent genes remain poised in the absence of Phc1
• Phc1 is essential for D-V patterning of eye development in mice

## Graphical abstract

**Figure:**
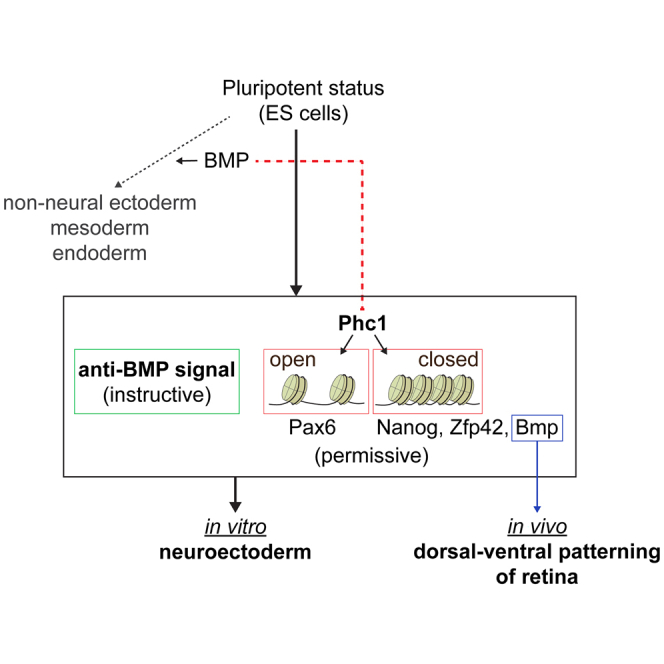

## Introduction

Neural induction, a process by which naive cells irreversibly acquire the neural cell fate, is one of the earliest events of embryogenesis.^1^^,^^2^ These neural cells then further differentiate into neurons or glial cells, which constitute the central and peripheral nervous systems.

The mechanisms of neural induction have been widely studied in amphibian embryos. Historically, the genes encoding neural inducers, *Noggin*, *Chordin*, and *Follistatin*, were isolated and shown to emanate from specific dorsal mesodermal tissue, or Spemann’s organizer.^3^^,^^4^^,^^5^ They directly bind to bone morphogenetic proteins, namely, BMP2/4/7, in the extracellular space and act as antagonists to block these BMPs from binding to the BMP receptor.^6^ Thus, the blockade of BMP signaling is essential to direct the cells into neural fate. This blocking event inhibits the phosphorylation of the carboxyl-terminal serine residues of the Smad1 protein, which is an intracellular mediator of the BMP signal, preventing the downstream genes of BMP signals from being activated. Instead, the inhibition of the BMP signal induces the expression of a series of transcription factors, which in turn activate the downstream transcriptional network to further promote neural differentiation. This is how the BMP signal affects the early ectodermal cells’ binary decision between the epidermis and neural fates.

In addition to the BMP antagonists, fibroblast growth factors (FGFs) also have neural inducing activity.^7^^,^^8^^,^^9^ One proposed mechanism is that FGF promotes the phosphorylation of the intermediate linker domain of the Smad1 protein, instead of its carboxyl-terminal domain, and restricts the Smad1 activity.^7^ In addition, a more recent model suggests that FGF signaling and the blockade of BMP act independently, with FGF directly inducing the neural genes.^8^^,^^9^ Together, the combination of BMP inhibitors and FGF is essential for directing naive cells toward the neural fate.

The above principle has been shown to be, at least in part, applicable to amniote embryos.^10^ In mouse embryos, Chordin and Noggin homologues emanate from the node, or the anterior portion of the primitive streak, and their compound mutants exhibit holoprosencephaly, or severe forebrain malformation at early embryonic stages,^11^^,^^12^ indicating a conserved mechanism for neural induction. However, the development of the posterior nervous system in the *Chordin*/*Noggin* double mutant mice is relatively normal, suggesting distinct mechanisms of neural induction from the amphibian embryos, where the entire neural induction is abolished. It has been recently demonstrated that the anterior and posterior neural cells are already separated at the epiblast stage, and this differentiation progresses independently.^13^^,^^14^

Likewise, neural differentiation from embryonic stem (ES) cells is severely perturbed when the cells are exposed to BMP signals.^15^^,^^16^^,^^17^ Moreover, FGF acts temporally during the early stages of neural induction,^17^^,^^18^^,^^19^^,^^20^ and the differentiation itself is perturbed when the signal is blocked.^18^^,^^21^^,^^22^ Therefore, the principle of the blockade of BMP in presence of FGF signal for neural fate determination is conserved in amniote embryos and their stem cells.^23^^,^^24^

While the cells start to express genes specific to the neural fate, the chromatin status, so-called the epigenetic status, characterized by histone and DNA modifications, dynamically changes. Pluripotent gene loci (e.g., *Nanog* and *Rex*) are usually open in the ES cell state, and some of them are bivalently associated with modification marks. During differentiation, these coding and flanking regions eventually condense to form heterochromatin, which is driven by DNA methylation and histone methylation or acetylation. Concurrently, the neural gene loci are gradually gaining open chromatin status.^25^^,^^26^^,^^27^

Polycomb group (PcG) proteins have been shown to influence these chromosomal dynamics^14^^,^^25^^,^^27^^,^^28^^,^^29^^,^^30^^,^^31^^,^^32^^,^^33^ and to regulate gene expression through chromatin modifications.^34^ PcG proteins form two major chromatin-modifying complexes, known as Polycomb repressive complexes 1 and 2 (PRC1 and PRC2). Each group is represented by different members of the PcG protein family, with core catalytic proteins, Ring1A/B for PRC1 and Ezh1/2 for PRC2.^35^ PRC1 monoubiquitinates histone H2A at lysine 119 (H2AK119ub), while PRC2 trimethylated histone H3 at lysine 27 (H3K27me3),^36^^,^^37^ both of which potentiate chromatin compaction. Cooperation between PRC1 and PRC2 complexes is essential for the regulation of gene expression.^38^^,^^39^ As the PRC1/PRC2 complexes dissociate from the target loci during lineage commitment, the developmental genes suppressed by the PRC1/PRC2 complexes in the pluripotent state are activated to promote differentiation.^38^^,^^40^ PRC1 is further categorized into canonical (cPRC1) and non-canonical (ncPRC1) PRC1, depending on the protein components that constitute the assembly.^33^^,^^41^ cPRC1 is involved in the PRC2-dependent recruitment of H3K27me3 via the Chromobox protein (Cbx), while ncPRC1 activity is mainly dependent on H2AK119ub but not PRC2 or H3K27me3.^42^^,^^43^

The dual roles of PcG complexes, either in activation or repression of the target genes, are achieved by the dynamic interaction of interacting partner proteins with the core proteins. Such cofactors include Polycomb Group Ring Finger protein (Pcgf),^44^ Cbx and Polyhomeotic Homologs (Phc), where Phc and Cbx join only the canonical type of PRC1, each with several paralogues, and the combination of these proteins changes during differentiation. For instance, the assembly’s initial constituents are Cbx7 and Pcgf6, whose expression is enriched in ES cells, and they are substituted with Cbx6/8 and Pcgf4, respectively, during neural differentiation.^40^^,^^45^^,^^46^ Moreover, Pcgf5 expression has been shown to increase as ES cells differentiate into neural progenitor cells (NPCs),^29^ and its loss prevents neural differentiation via the aberrant activation of the SMAD2/TGF-β signaling pathway.^29^ Similarly, other PRC1 components, including Cbx6, Cbx8, Cbx3, Rybp, and Auts2, are enriched in NPCs,^40^^,^^47^^,^^48^ and mice harboring some of these mutant genes fail to develop properly with a defect in neural differentiation. Together, the differentiation process should be correlated with inductive molecules and chromatin dynamics. However, the molecular mechanisms by which the epigenetic changes of PRC1 are regulated during neural differentiation have not been fully understood.

In this study, we attempted to understand the roles of the PRC in the regulation of stem cells and neural differentiation. We took advantage of the neural differentiation system of ES cells and identified *Phc1* (also known as *Rae28/Retinoic acid early response gene*) as one of the genes whose expression is downregulated by the BMP signal.

We generated mutant stem cell lines with the CRISPR/Cas9 system and discovered that *Phc1* is essential for early neural differentiation. We further conducted genome-wide expression profiling and chromatin accessibility assays. We demonstrated that the epigenetic modification regulated by *Phc1* controls the initiation of differentiation.

## Results

### BMP treatment regulates the gene expression of PRC components

**Figure 1:**
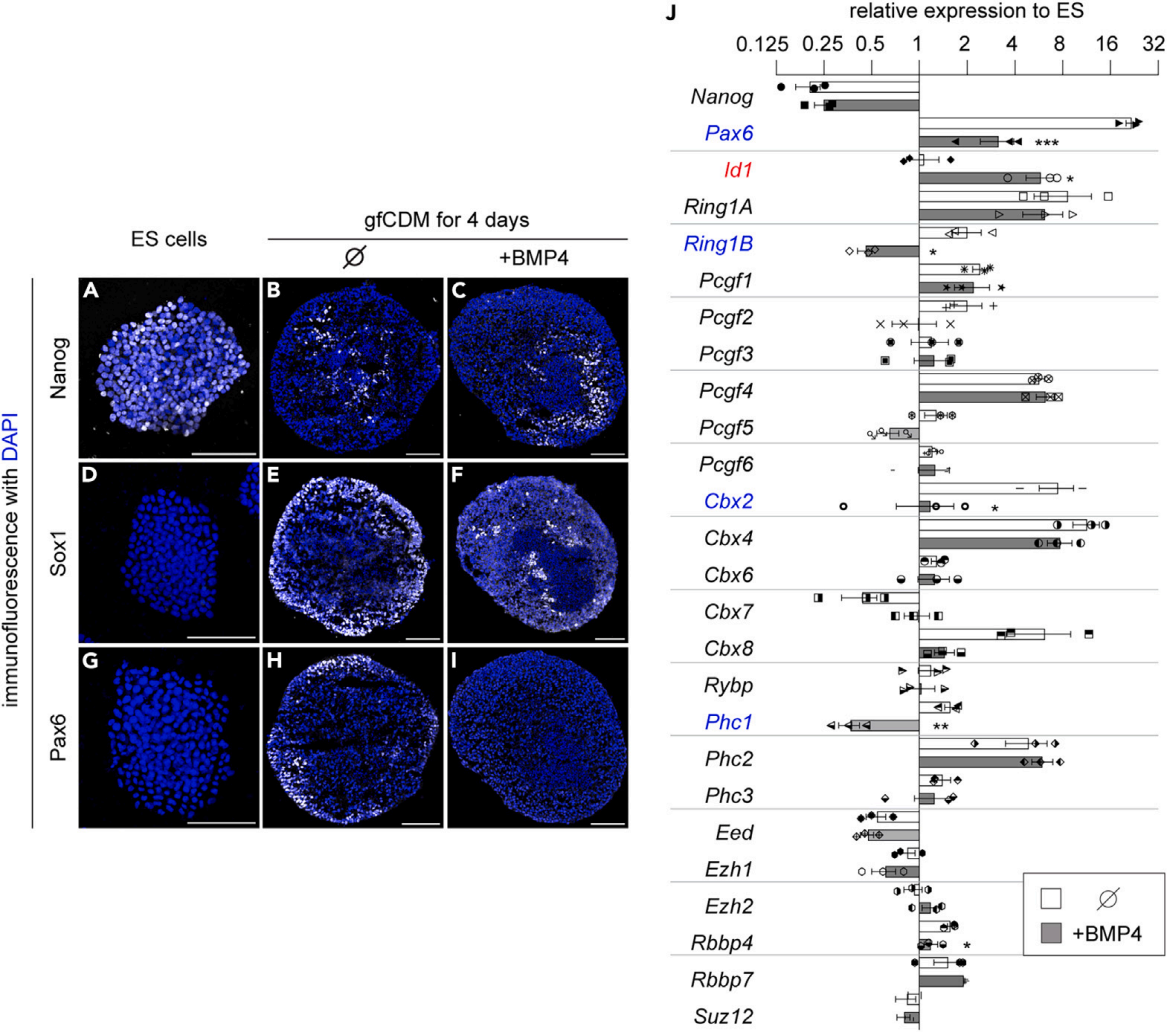
The differentiation of ES cells into non-neural cells downregulated some of the PRC genes (A–I) Immunostaining of ES cells (A, D, G) and cells differentiated in gfCDM (B, C, E, F, H, I), either without (B, E, H) or with 10 ng/mL BMP4 (C, F, I), for four days with anti-Nanog (A–C), Sox1 (D–F) and Pax6 (G–I) antibodies. Scale bars = 100 μm. (J) RT-qPCR analysis of the PRC genes, whose expression levels are relative to those in the ES cells, at day 4 differentiated in gfCDM without or with 10 ng/mL of BMP4. Genes that were upregulated and downregulated by the BMP4 treatment are indicated by red and blue characters, respectively. Data are represented as mean ± SEM. Statistical differences were calculated using two-tailed Student’s *t* test. ∗ indicate statistically significant p < 0.05, ∗∗ indicate statistically significant p < 0.01 and ∗∗∗ indicate statistically significant p < 0.001.

To understand the relationships between neural inducing signals and PRC functions during neural differentiation, we took advantage of the *in vitro* neural differentiation system of mouse ES cells. Herein, we differentiated ES cells into forebrain-type neural progenitor cells by using a three-dimensional differentiation system. The differentiation medium, namely, growth factor-free chemically defined differentiation medium (gfCDM),^49^^,^^50^^,^^51^ which contains no unidentified growth factors to which the effects of exogenously treated growth factors can be easily evaluated. With this differentiation protocol, Nanog expression was drastically reduced (Figures 1A and 1B) compared to that in ES cells four days after the start of differentiation (hereafter denoted as day 4), suggesting that the cells entered the somatic cell state. Instead, these cells exhibited early neural cells characterized by Sox1 and Pax6, which were not present in the pluripotent state (Figures 1D, 1E, 1G, and 1H).

Next, we attempted to address what occurs when signals that divert the cells into a non-neural state are present and focused on the effect of BMP4. Upon treatment with BMP4, the Nanog expression was comparable to that of the control differentiated cells (Figures 1B and 1C), indicating that the treatment in this differentiation protocol has no impact on the differentiation itself. However, the number of cells positive for Sox1 and Pax6 was markedly decreased (Figures 1F and 1I), suggesting that BMP4 inhibits neural differentiation.^14^^,^^17^^,^^52^

We reasoned that treatment with BMP promoted differentiation into other lineages. We thus investigated the expression of *Pax6* for neural ectoderm and *Id1*^53^ for non-neural ectoderm. As a result, BMP4 treatment upregulated *Id1* while decreasing *Pax6* expression (Figure 1J). This observation validated the effect of BMP4, and showed that the differentiating ES cells exposed to BMP signaling were diverted from neural fate commitment (Figure 1J).

We next examined the gene expression of the PRC components with RT-qPCR. While most genes were not influenced by BMP treatment during neural differentiation, we found a decrease in the expression of *Ring1B*, *Cbx2*, and *Phc1* (Figure 1J).

Therefore, the results suggested that the PRC genes are transcriptionally regulated by signaling molecules, and further raised the possibility that the PRC subunits dynamically change during neural differentiation.

### Phc1, whose function is replaceable with that of Phc2, is essential for neural differentiation

We next sought to address the essential roles of PRC component factors in neural development, whose expression was analyzed and altered when exposed to BMP signaling. We established stem cells deficient in the above genes by means of CRISPR/Cas9 mutagenesis.

The *Ring1B*-knockout (*Ring1B*-KO; Figure S1A) exhibited spontaneous differentiation (Figures S1B and S1C), and as seen in the RT-qPCR, the upregulation of differentiated genes was consistent with previous observations (Figure S1D).^54^ In contrast, *Cbx2*-knockout cells (*Cbx2*-KO; Figure S1E) did not display any explicit phenotypes in terms of neural differentiation at day 4. Moreover, when the differentiation was extended until day 7 with ChIR99021 treatment from day 4 onwards, which prompted the formation of retinal-like structure,^55^ the cells showed similar evaginations as the wild-type cells, with significant expression of Sox1 and Pax6. Rax, which defines retinal identity, was confirmed as represented by GFP (Figures S1F–S1M). This observation suggests that compensatory mechanisms exist among other paralogues.

**Figure 2:**
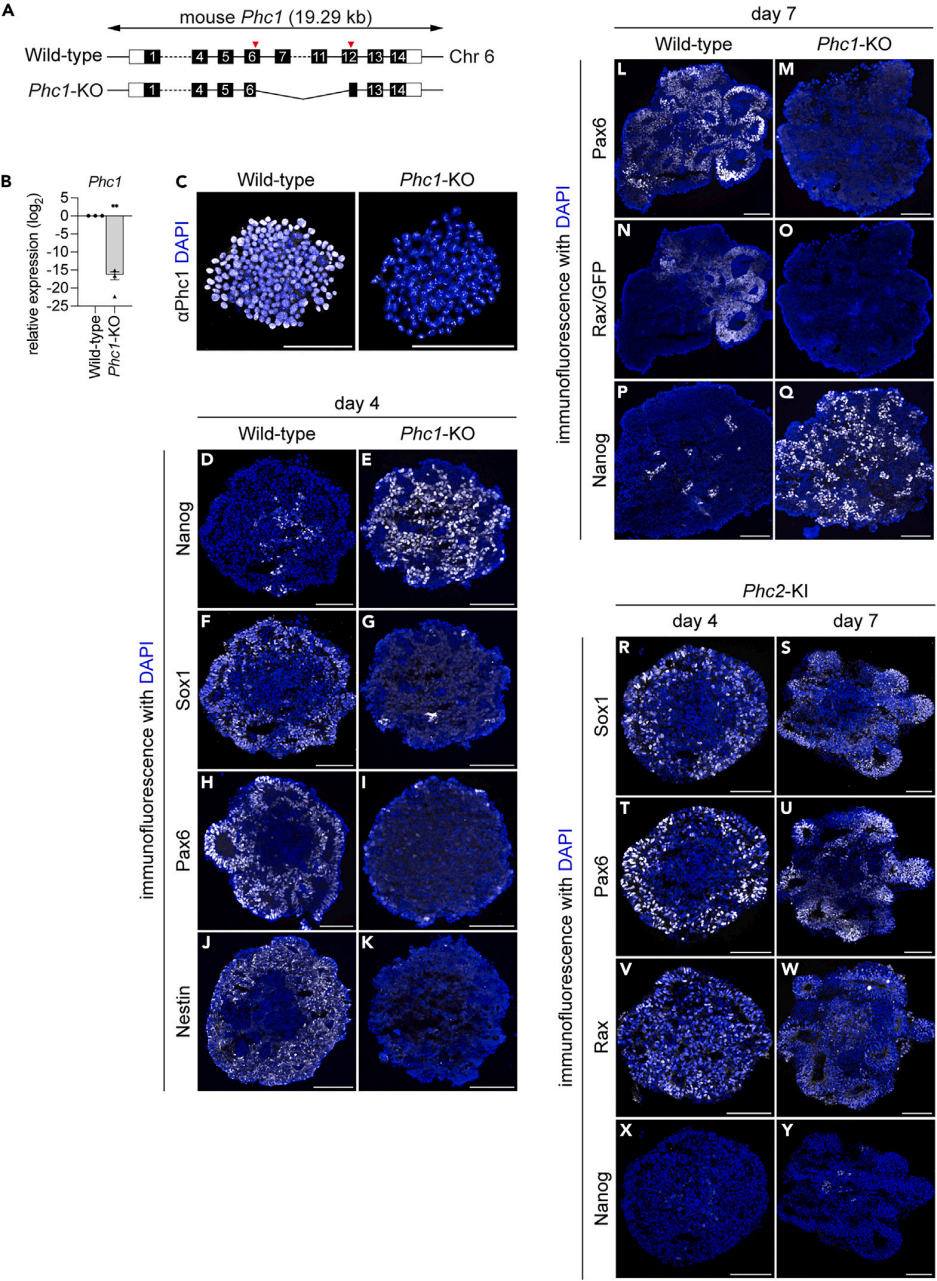
*Phc1* is required for early neural differentiation (A) Schematic representation of the *Phc1*-KO cells. Two guide RNAs were designed at exons 6 and 12 of the *Phc1* gene locus on chromosome 6 (Chr6) (red arrowheads). (B and C) RT-qPCR (B) and immunofluorescence (C) analyses showed that *Phc1* expression was completely abolished in *Phc1*-KO cells. Note that the signals found in the *Phc1*-KO are non-specific. (D–K) *Phc1*-KO cells fail to differentiate into early neural cells. Wild-type (D, F, H, J) and *Phc1*-KO (E, G, I, K) cells differentiated with gfCDM/MG for four days were analyzed with anti-Nanog (D, E), Sox1 (F, G) Pax6 (H, I) and Nestin (J, K) antibodies. (L–Q) *Phc1*-KO cells fail to acquire retinal identity. Wild-type (L,N,P) and *Phc1*-KO (M,O,Q) cells were further differentiated with ChIR99021 to day 7 and were analyzed with anti-Pax6 (L,M) GFP (for Rax) (N,O) and Nanog (P,Q) antibodies. (R–Y) Phc2 can replace Phc1. The *Phc2*-knock-in cell line was established as in Figure S2D. The ES cells were differentiated for four (R, T, V, X) or seven (S, U, W, Y) days and were analyzed with anti-Sox1 (R, S), Pax6 (T, U) Rax (V, W) and Nanog (X, Y) antibodies. Scale bars in (C-Y) = 100 μm. Data are represented as mean ± SEM. Statistical differences were calculated using two-tailed Student’s *t* test. ∗∗ indicate statistically significant p < 0.01.

As for another candidate gene Phc1, the *Phc1*-knockout (*Phc1*-KO; Figure 2A), which did not express Phc1 as validated by RT-qPCR (Figure 2B), immunofluorescence (Figure 2C) and Western blot (Figure S2D), showed a distinct phenotype with a significantly higher number of Nanog-positive cells compared to the wild-type at day 4 (Figures 2D and 2E). Conversely, the expression of Sox1 (Figures 2F and 2G), Pax6 (Figures 2H and 2I) and Nestin (Figures 2J and 2K) was greatly downregulated. Furthermore, according to the extension of the differentiation until day 7 with ChIR99021 at day 4 onwards, Pax6 (Figures 2L and 2M) and Rax (Figures 2N and 2O) were still not expressed in the *Phc1*-KO cells, with abundant Nanog expression (Figures 2P and 2Q). Treatment with SAG on day 3 was carried out to attempt ventral diencephalic differentiation,^49^^,^^51^ but the *Phc1*-KO cells did not express any early hypothalamus cells positive for Nkx2.1 at day 7 (Figures S2A and S2B). Together, these findings suggest that *Phc1*-KO cells have essentially lost the competence to initiate neural differentiation and essentially abandoned neural differentiation.

To validate the specific phenotypes observed in the *Phc1*-KO cells, we introduced the full coding region of *Phc1* containing the sequence encoding the Hemagglutinin (HA)-tag at the starting site of the *Phc1* genomic locus, and employed homologous recombination and the CRISPR/Cas9 method to generate *Phc1*-knockin (*Phc1*-KI) cells (Figures S2C and S2D). As a result, the reverted expression of Sox1, Pax6 and Rax and fewer Nanog-positive cells were confirmed at day 4 and 7 in the *Phc1*-KI line in the *Phc1*-KO background (Figures S2E–S2L). Thus, the phenotypes described earlier in the mutant (*Phc1*-KO) cells were caused by the specific loss of the *Phc1* gene, but not by the mutations randomly introduced in another locus of the genome.

Interestingly, the neural differentiation was also rescued by knocking-in *Phc2*,^56^ a paralog of Phc1, into the *Phc1*-KO cells (Figures S2C and S2D), revealing reverted Sox1, Pax6 and Rax expression (Figures 2R–2W) and reduced Nanog expression (Figures 2X and 2Y). This finding suggests that Phc1, which has structural domains similar to those of Phc2, is replaceable by Phc2 for its function.

We further investigated the characteristics of the *Phc1*-KO ES cells. This mutant cell was able to be maintained normally like the wild-type cells, with comparable expression of *Nanog*, *Sox2*, *Pou5f1/Oct4*, *Klf4* and *Zfp42*/*Rex1*, showing characteristics of ES cells (Figures S3A–S3D), while *Hoxa1*, *Hoxa3* and *Foxg1* were upregulated in the *Phc1*-KO, as reported^57^^,^^58^ (Figure S3A), which is consistent with the observation that Phc1 is required for suppressing the Hox gene expression.^56^

We further examined whether cell proliferation changes. We performed a 5-ethynyl-2′-deoxyuridine (EdU) incorporation assay and counted the S-phase cells, and immunofluorescence with pHH3 antibody was carried out to detect the M-phase cells. Both the wild-type and *Phc1*-KO ES cells exhibited similar numbers of positive cells in both assays (Figures S3E–S3J), suggesting that the proliferation rate is comparable in both genotypes, with no significant changes in phenotype,^59^ regardless of the upregulation of some of the *Hox* genes (Figure S3A). Thus, the *Phc1*-KO ES cell characteristics appear to be indistinguishable from those of wild-type ES cells.

Together, Phc1 is dispensable for the maintenance of ES cells, but is required for the cells to exit the pluripotent state and enter the neural fate commitment.

### *Phc1*-KO cells undergoing neural differentiation remain in a pluripotent state

To compare the gene expression of wild-type and *Phc1*-KO neural progenitor cells in a genome-wide manner, we conducted expression profiling by means of mRNA sequencing (mRNA-seq).

Wild-type and *Phc1*-KO cells were differentiated for four days with the gfCDM/Matrigel protocol (gfCDM/MG), and the cells were subjected to mRNA-seq. In this expression profiling, two independently established mutant clones were analyzed (Figure S4 for the analysis of another clone).

**Figure 3:**
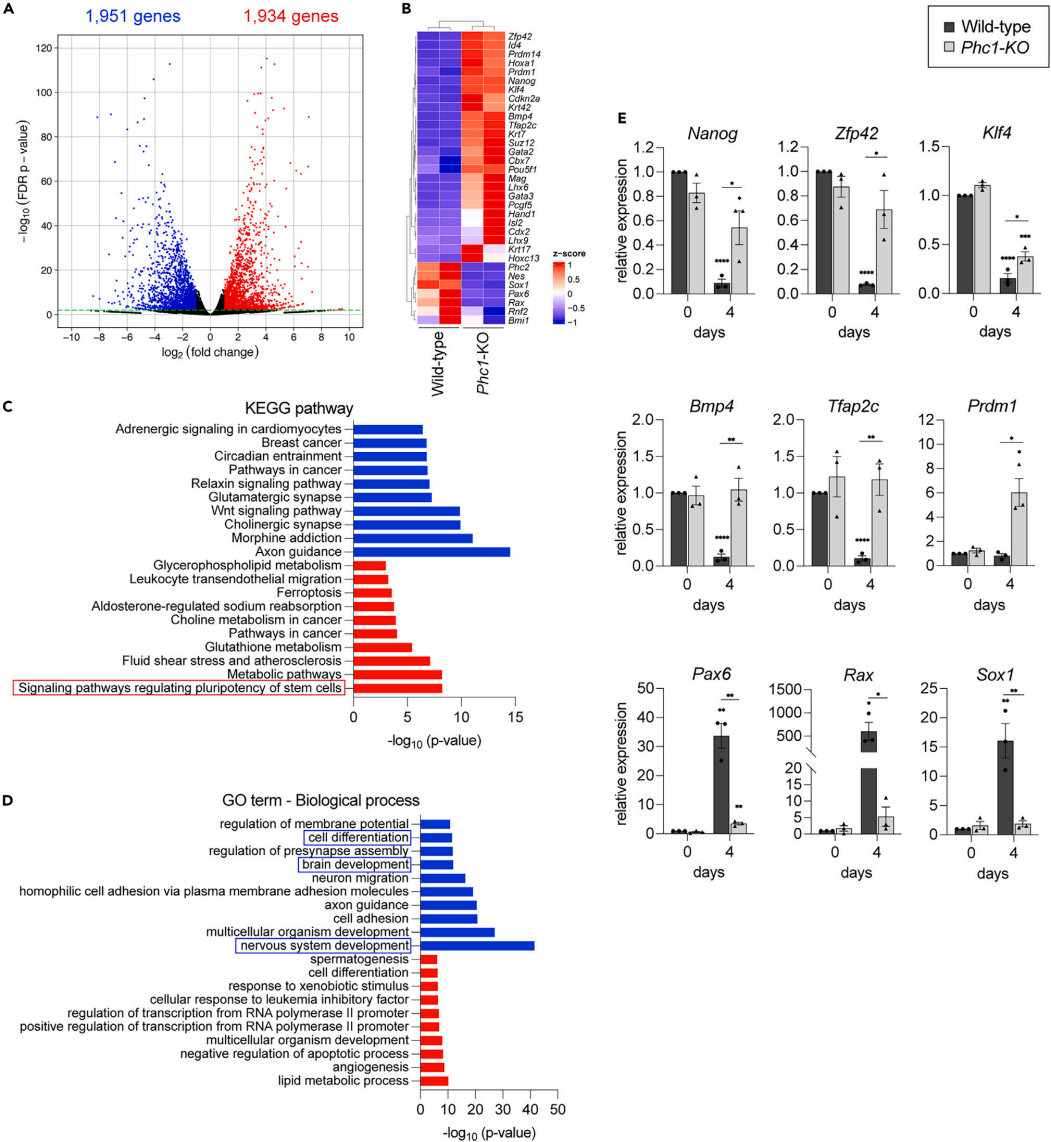
Pluripotent genes are aberrantly upregulated in *Phc1*-KO neural progenitor cells during neural differentiation (A) The *Phc1*-KO cells cultured with gfCDM for four days failed to acquire neural progenitor cell identity. In the volcano plot, the genes whose expression levels were different by more than 2-fold with FDR-adjusted p < 0.01 are colored in red (1,934 genes upregulated in *Phc1*-KO) and blue (1,951 genes downregulated in *Phc1*-KO). Detailed information (gene list) is provided in Table S2. (B) Heatmap representation of the expression of representative genes in wild-type and *Phc1*-KO. (C and D) KEGG pathway (C) and GO biological process (D) enrichment analysis of upregulated (red) or downregulated (blue) genes in *Phc1*-KO. (E) RT-qPCR analysis of wild-type and *Phc1*-KO cells at day 0 and day 4 for the indicated genes. Data are represented as mean ± SEM. Statistical differences were calculated using two-tailed Student’s *t* test. ∗ indicate statistically significant p < 0.05, ∗∗ indicate statistically significant p < 0.01, ∗∗∗ indicate statistically significant p < 0.001 and ∗∗∗∗ indicate statistically significant p < 0.0001.

As a result, the mRNA levels of 1,951 genes (indicated with blue dots; Figure 3A) were enriched in the wild-type cells, while 1,934 genes (indicated with red dots; Figure 3A) were aberrantly upregulated in the *Phc1*-KO cells with false discovery rate (FDR)-adjusted p values less than 0.01. Consistent with previous findings (Figure 2), *Sox1*, *Pax6*, *Nestin* and *Rax* expression was significantly downregulated in the *Phc1*-KO cells, confirming the essential roles of Phc1 in early neural differentiation (Figure 3B).

On the other hand, the expression of genes characterizing pluripotency was higher in the *Phc1*-KO cells (Figure 3B). For instance, *Nanog*, *Klf4*, *Pou5f1*, and *Zfp42*, which are expressed specifically in ES or epiblast cells, were enriched in the *Phc1*-KO cells (Figure 3B). Moreover, some of the trophoblast genes *Hand1*,^60^ *Cdx2*, *Krt7*,^61^ *Tfap2c*^62^ and *Prdm1*/*Blimp1*^63^ also showed higher expression levels compared to those in the wild-type (Figure 3B), suggesting that some cells exit the pluripotent state but are directed to the non-neural fate instead. Notably, these trophectoderm genes are associated with the BMP signaling pathway,^61^^,^^64^ and consistently, *Bmp4* expression was upregulated in *Phc1*-KO cells. A pathway analysis suggested that the signaling pathways regulating the pluripotency of stem cells were significantly upregulated (KEGG pathway; Figure 3C), while genes for brain and neural/neuronal differentiation (Gene ontology biological process; Figure 3D) were downregulated in *Phc1*-KO cells. The same expression patterns were found in another *Phc1*-KO clone (Figure S4; Table S3), validating the reproducibility of the phenotype.

Next, we used RT-qPCR to further investigate the temporal changes in gene expression. Both wild-type and *Phc1*-KO cells exhibited comparable expression of the pluripotent genes *Nanog*, *Zfp42* and *Klf4* at the pluripotent stage. At day 4, the levels remained greater in *Phc1*-KO cells, while these genes were found to decrease in wild-type cells (Figure 3E). In addition, we found that the expression of the trophectoderm genes *Bmp4*, *Tfap2c*, and *Prdm1* at day 4 in wild-type cells was significantly lower than that at day 0, but it remained higher in *Phc1*-KO cells (Figure 3E). Conversely, the early neural genes *Pax6*, *Rax* and *Sox1* drastically increased in the wild-type cells at day 4, but not in the KO cells (Figure 3E). Therefore, the expression analysis suggests that the *Phc1*-KO cells are closer to the pluripotent state.

To systematically determine if the *Phc1*-KO cells are close to the pluripotent state, we analyzed the publicly available transcriptome data of ES and neural progenitor cells,^65^ and performed a comparative analysis with our data on wild-type and *Phc1*-KO neural progenitor cells. We compared the quantitative differences of all the gene expression levels of day 4 neural progenitor cells versus ES cells and *Phc1*-KO cells against wild-type cells. We found a tendency toward an inverse correlation (Figure S6A; Pearson’s r score = −0.420). Therefore, the idea that *Phc1*-KO cells are in a state close to pluripotent cells was further substantiated by this analysis.

The mRNA-seq data also exhibited aberrant upregulation of some Hox genes (*Hoxa1* and *c13*) in the *Phc1*-KO cells (Table S2). In addition, in the *Phc1*-KO embryos, the perturbation of the thoracic identities has been observed,^56^^,^^66^ raising the possibility that Phc1 plays a role in determining posterior identities during neural differentiation.

To address this possible role, we made use of a protocol for posterior neural differentiation. We differentiated ES cells with an N2/B27-based medium treated with ChIR99021 and RA/SAG^67^ (Figure S5A; see STAR Methods for details). In wild-type cells, Rax expression, which can be found only in the anterior levels, decreased by a treatment with ChIR (Figures S5B and S5C), suggesting that the anterior and posterior neural differentiation was properly achieved by this protocol. The comparable Pax6 expression in both differentiation conditions, and the absence of Nanog confirmed the neural differentiation (Figures S5D–S5G).

In contrast, the *Phc1*-KO cells failed to express Rax and Pax6 in both conditions (Figures S5H–S5K), suggesting the neural differentiation itself was perturbed. In addition, the Nanog-positive cells aberrantly increased (Figures S5L and S5M), suggesting Phc1 is required not only for anterior but also for posterior neural differentiation.

We investigated the expression of additional genes by RT-qPCR. The expression of *Otx2*, which characterizes early forebrain/midbrain cells, was significantly upregulated during differentiation with control N2/B27 medium and decreased by treatment with ChIR. Conversely, the expression of posterior genes *Hoxa3*, *b6* and *b9* increased, suggesting that the posteriorization was properly achieved by this protocol (Figure S5N).

In contrast, *Phc1*-KO cells exhibited lower *Otx2* and higher *Nanog* expression than wild-type cells, suggesting that early neural differentiation is, at least partially, perturbed. Moreover, the mutant cells were less responsive to ChIR treatment, and all tested genes *Hoxa3*, *b6* and *b9* expression were significantly downregulated. Therefore, posterior neural differentiation was entirely disrupted in *Phc1*-KO cells, and the Hox genes were expressed randomly.

Taken together, the *Phc1*-KO cells in an attempt to differentiate into neural cells are either at pluripotent or early non-neural stages, resulting in failure to convert into neural cells.

### Phc1 is dispensable for mesoderm and endoderm differentiation

From the above experiments, it is evident that Phc1 plays a role in early neural development. To investigate whether Phc1 is required for differentiation into other germ layers, we generated embryoid bodies (EBs), where both wild-type and *Phc1*-KO cells were induced to randomly differentiate into all three germ layers.

**Figure 4:**
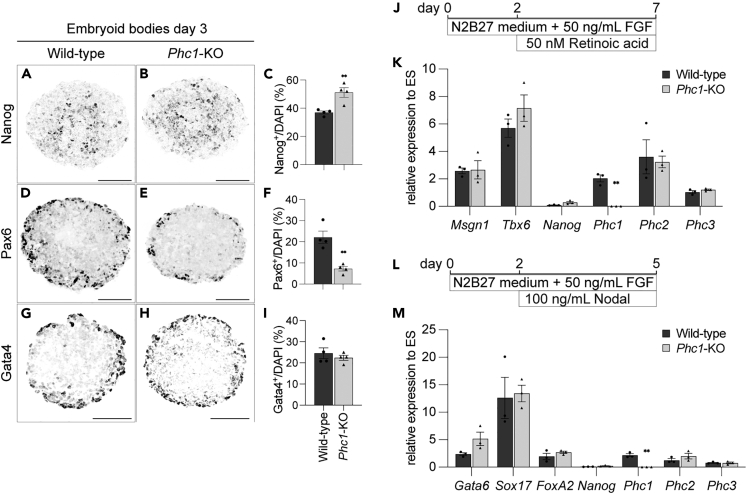
Phc1 is dispensable for mesoderm and endoderm differentiation (A–I) Formation of embryoid bodies from wild-type (A, D, G) or *Phc1*-KO (B, E, H) ES cells for three days. The expression of Nanog (naive cells; A-C), Pax6 (neuroectoderm; D-F) and Gata4 (meso- and endoderm cells; G–I) was analyzed by immunofluorescence. Scale bars = 100 μm. (C, F, and G) Quantification of the cells positive for each gene. (J–M) Schematic representation of mesoderm (J) and endoderm (L) differentiation and RT-qPCR analysis of the marker genes (K, M). Data are represented as mean ± SEM. Statistical differences were calculated using two-tailed Student’s *t* test. ∗∗ indicate statistically significant p < 0.01.

After three days of differentiation, the number of Nanog-positive cells was higher in the *Phc1*-KO (Figures 4A–4C) than in the wild-type cells, as in the case where the cells were differentiated with gfCDM (Figure 2E). However, the rate of positive cells in the *Phc1*-KO cells (Figures 4B and 4C) was significantly lower than that in the cells differentiated with gfCDM (Figure 2E). As the differentiation medium for EBs contains unidentified growth factor(s), it was assumable that the cells bypassed Phc1 in repressing the *Nanog* gene. Thus, we asked whether neural differentiation is still dependent on Phc1 under these differentiation conditions. As a result, we found fewer Pax6-positive neuroectodermal cells in *Phc1*-KO cells than in wild-type cells (Figures 4D–4F), which was consistent with the differentiation by gfCDM (Figures 2H and 2I). In contrast, Gata4 expression, which characterizes mesoderm differentiation,^68^ was comparable in both genotypes (Figures 4G–4I), suggesting that mesoderm differentiation is less dependent on Phc1 function.

This result prompted us to ask about the possibility that mesoderm and endoderm differentiation were less affected by the attenuation of the *Phc1* gene. To address this hypothesis, we established directed differentiation protocols for mesoderm and endoderm lineages. ES cells were treated with basic FGF (bFGF/FGF2) and retinoic acid (RA) for seven days to induce mesoderm differentiation, based on the published protocol with some modifications^69^ (Figure 4J; see STAR Methods for details). The expression of the presomitic paraxial mesodermal genes *Msgn1* and *Tbx6*^70^ was upregulated, with downregulated *Nanog* expression, and these expression levels were similar in both cells (Figure 4K).

Similarly, endoderm differentiation was achieved with the combination treatment of bFGF and Nodal^71^ (Figure 4L). The expression of the early endodermal genes *Gata6*, *Sox17* and *Foxa2* at day 5 was comparable in the wild-type and *Phc1*-KO cells (Figure 4M). Moreover, there were no distinct changes in the expression of *Phc2* and *Phc3* under both differentiation conditions (Figures 4K and 4M), indicating that the regulation of the expression of Phc paralogues is independent of each other.

Our findings, therefore, indicate that *Phc1*-KO cells still possess the competence to differentiate into the other two germ layers of the mesoderm and endoderm.

### The chromatin loci of the pluripotent genes remain poised during an attempt at neural differentiation

**Figure 5:**
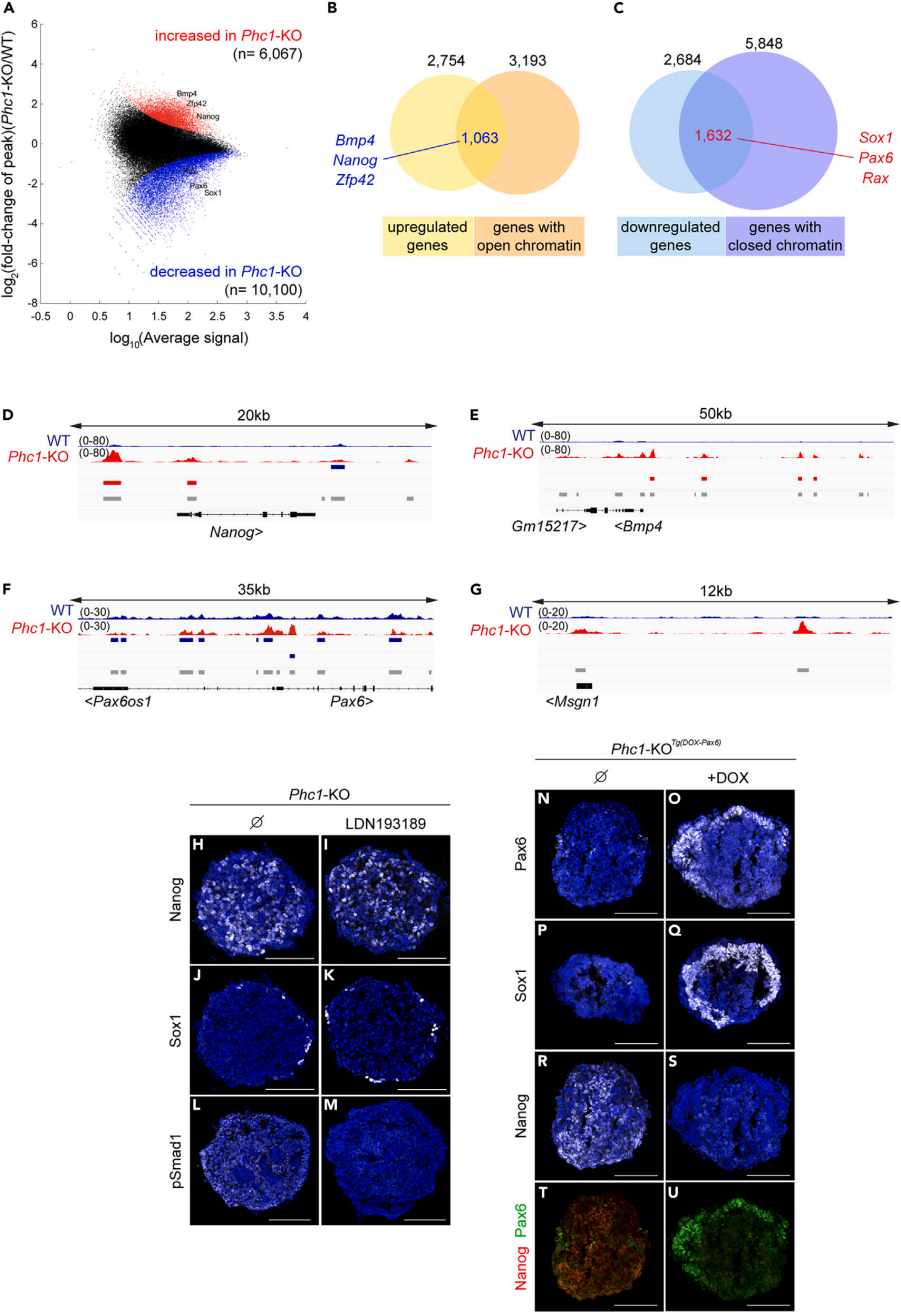
Phc1 is required for chromatin compaction during neural differentiation (A) The peaks whose signals were significantly different (FDR-adjusted p < 0.01) are colored with red (more accessible in the *Phc1*-KO) and blue (less accessible in the *Phc1*-KO). The gene name indicates the peak whose p value was lowest among the peaks within 50kb of the gene. (B and C) The number of genes that were upregulated (B) or downregulated (C) in the *Phc1*-KO and genes that were more accessible (B) and less accessible (C) in the *Phc1*-KO. (D–G) ATAC-seq peak tracks of WT (wild-type) and *Phc1*-KO cells at day 4 of gfCDM differentiation at the *Nanog* (D), *Bmp4* (E), *Pax6* (F) and *Msgn1* (G) loci. (H–M) The failure of neural differentiation in *Phc1*-KO cells cannot be rescued by treatment with the BMP inhibitor LDN193189, as shown by immunofluorescence with anti-Nanog (H, I), Sox1 (J, K) and pSmad1 (L, M) antibodies. (N–U) Timed induction of Pax6 rescued the *Phc1*-KO phenotype in terms of neural induction. *Phc1*-KO^*Tg*(*DOX-Pax6*)^ cells were differentiated for four days with gfCDM, either without (N, P, R, T) or with doxycycline (O, Q, S, U) from day 3 onwards, and analyzed with anti-Pax6 (N, O), Sox1 (P, Q) and Nanog (R, S) antibodies. Merger images are in (T, U). Scale bars in (H-U) = 100 μm.

As Phc1 is a component of the PcG complex, which drives essential modifications on chromatin, we then sought to analyze the chromatin accessibility of day 4 neural progenitor cells, both wild-type and *Phc1*-KO cells, with Assay for Transposase-Accessible Chromatin with high-throughput sequencing (ATAC-seq) (Figure 5A).

Our results revealed increased genomic accessibility in the *Phc1*-KO cells, especially in the pluripotent genes *Nanog* and *Zfp42,* where more peaks were found within or around the locus (Figure 5A). This suggests that overall chromatin compaction failed in *Phc1*-KO neural progenitor cells.

To determine if there is a correlation between the gene expression and the accessible chromatin status or peaks, we compared the mRNA-seq (Figures 3A and 3B) and ATAC-seq data and found a positive correlation (p value <10e^−10^) with a Pearson’s r score of 0.258 (Figure S6B). In detail, among the 3,193 genes with peaks that were significantly (adjusted p value <0.01) accessible in *Phc1*-KO, 1,063 genes (33.3%) were upregulated in the *Phc1*-KO cells (Figures 3A and 5B; Table S2). On the other hand, of the 5,848 genes with less accessible peaks in the *Phc1*-KO, 1,632 genes (27.9%) had reduced expression compared to the wild-type (Figures 3A and 5C; Table S2), including the early neural genes *Pax6* and *Rax*. Thus, chromatin status and gene expression are greatly dependent on each other.

A detailed analysis of the pluripotency gene *Nanog* revealed two regions that are more accessible in its flanking region in the *Phc1*-KO cells (Figure 5D). The *Bmp4* gene also had four accessible peaks in *Phc1*-KO (Figure 5E). In contrast, the *Pax6* gene had eight peak regions that were more accessible at its flanking region in the wild-type (Figure 5F). These accessible sites corresponded to higher *Nanog* and *Bmp4* expression and lower *Pax6* expression in *Phc1*-KO (Figure 3B). In contrast, there were no significant changes in accessible peak regions around the mesoderm differentiation gene *Msgn1* (Figure 5G), which is consistent with the results that mesoderm and endoderm differentiation were rather intact upon lacking Phc1 function (Figures 4K and 4M). Therefore, Phc1 is required for chromatin compaction around pluripotent genes to suppress their expression, and thereby confer a permissive effect on the cells to differentiate into the neural cell fate.

Because the *Bmp4* gene locus has more accessible regions (Figure 5E) and was highly expressed at day 4 in *Phc1*-KO cells (Figure 3B), we hypothesized that blocking the BMP signal may rescue the *Phc1*-KO phenotype of failure to differentiate into neural cells. To test this hypothesis, we differentiated the cells with the BMP antagonist LDN193189 for four days. However, the cells failed to differentiate into early neural cells, with abundant Nanog (Figures 5H and 5I) and little to no Sox1 (Figures 5J and 5K)-expressing cells, albeit the BMP signal was successfully blocked, as confirmed by staining with phosphorylated Smad1 (pSmad1), which reflects the activated BMP signal (Figures 5L and 5M). Therefore, epigenetic regulation by Phc1 is an essential downstream step for neural induction combined with anti-BMP signaling.

Next, we sought to rescue the *Phc1*-KO phenotype by exogenous introduction of the *Pax6* gene, as Pax6 is the determinant of neuroectodermal cell fate.^72^ We generated a transgenic cell line carrying the inducible *Pax6* transgene in *Phc1*-KO (*Phc1*-KO^*Tg*(*DOX-Pax6*)^). Without treatment, these cells differentiated with gfCDM for four days and behaved like *Phc1*-KO cells, with no neuroectodermal differentiation, and Nanog remained highly expressed (Figures 5N, 5P, and 5R). However, when the cells were treated with doxycycline (DOX) from day 3 onwards, both neuroectodermal genes Pax6 and Sox1 were expressed at day 4 (Figures 5O and 5Q), and Nanog expression reciprocally decreased (Figures 5S–5U). These findings indicate that Pax6 represses pluripotent genes and promotes neural differentiation despite the absence of Phc1 function.

### BMP4 signaling is perturbed in the developing eye field of *Phc1* homozygotic embryos

While *Phc1* is essential for early neural differentiation in the ES cell experimental system, embryos deficient in the *Phc1* gene are not affected at early embryogenesis.^56^^,^^66^ Instead, *Phc1*-KO (*Phc1*^*−/−*^) embryos survive until the perinatal stage with defects in anterior-posterior skeletal arrangements, eye-field formation, internal organs and heart,^66^ and they die shortly after birth because of heart defects. The apparent phenotypic discrepancies found in the *in vitro* ES cell differentiation system (Figures 1, 2, 3, 4, and 5) and those in the *in vivo Phc1*-KO embryos^56^^,^^66^^,^^73^^,^^74^ were presumably caused by the partially common yet diverse mechanisms between both experimental systems. We thus sought to explore the common mechanisms and describe the differences between the two experimental systems.

**Figure 6:**
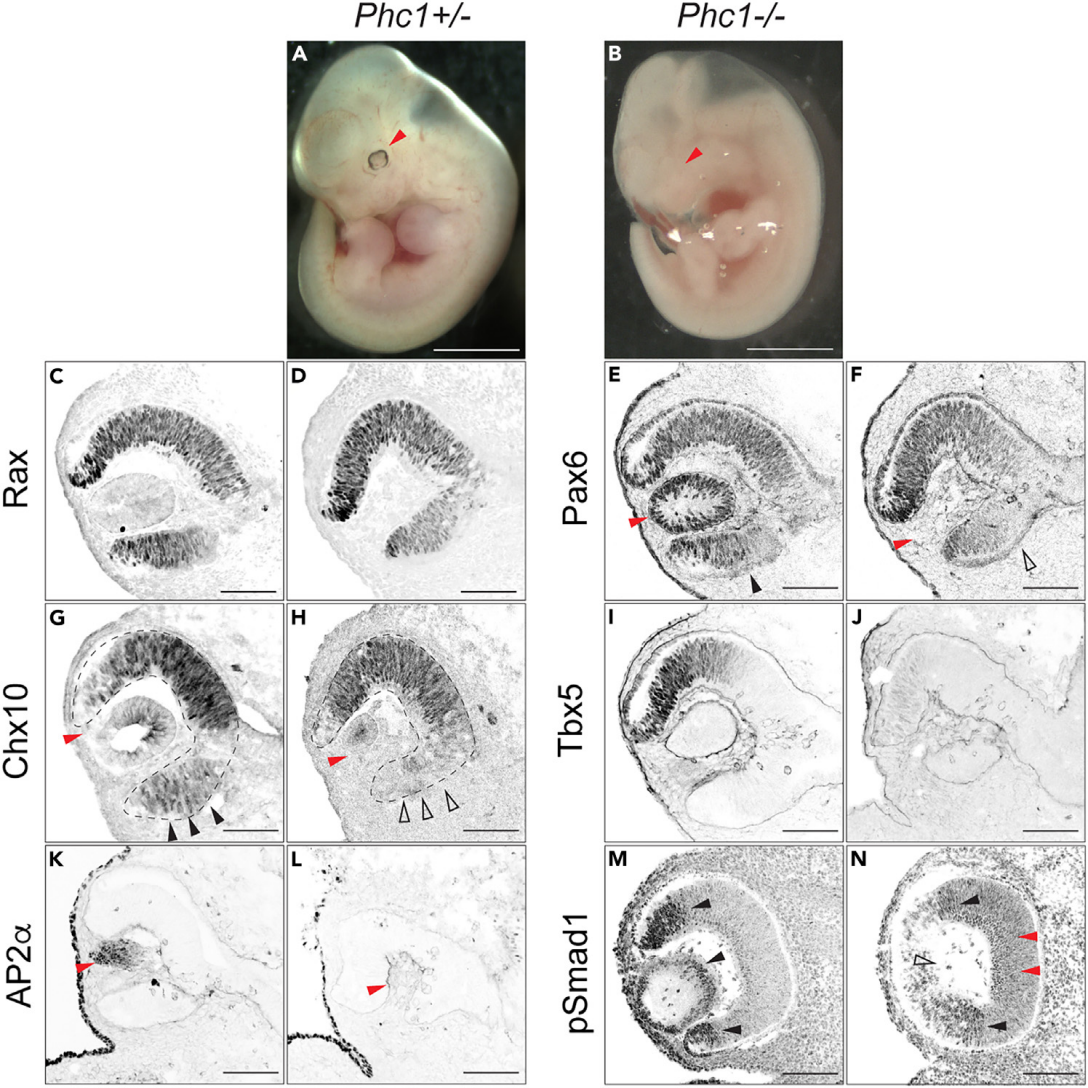
Phc1 is required for establishing the dorsal-ventral polarity of the neural retina (A and B) Gross appearance of the heterozygote (A; *Phc1*^*+/−*^) and homozygotic (B; *Phc1*^*−/−*^) e11.5. Note that the tail tip of the *Phc1*^*−/−*^ embryo was cut before taking pictures. (C–N) Immunofluorescence analysis of retinal sections of e11.5. *Phc1*^*+/−*^ (C,E,G,I,K,M) and *Phc1*^*−/−*^ (D,F,H,J,L,N) retinas were analyzed with anti-Rax (C,D), Pax6 (E,F) Chx10 (G,H), Tbx5 (I,J) AP2α (K,L) and pSmad1 (M,N) antibodies. Positive cells and negative cells for staining are indicated by filled and open black arrowheads, respectively. Lens areas are indicated by red arrowheads. Scale bars in (A,B) = 1 mm and (C–N) = 100 μm.

At embryonic day 11.5 (e11.5), the trunk structure was essentially indistinguishable except for the size being smaller in the homozygous mutant than in its heterozygous littermate (Figures 6A and 6B). However, evident retinal hypoplasia was discovered (Figures 6A and 6B; red arrowheads). According to the sectional analysis, Rx and Pax6 were similarly expressed in the dorsal and ventral parts of the *Phc1*^*+/−*^ retina (Figures 6C and 6E). In the homozygous mutant retina, Pax6 expression was still present (Figures 6D and 6F); however, ventral Pax6 expression was weakened (Figure 6F; open arrowhead), suggesting that dorsal-ventral (D-V) polarity in the retina was somehow perturbed in the homozygous mutants. This observation was also evident in another retinal marker, Chx10, where ventral expression was severely diminished (Figures 6G and 6H). The dorsal part, characterized by Tbx5 expression, was also affected by the *Phc1* homozygotic mutation (Figures 6I and 6J). Therefore, Phc1 is required for the establishment of the dorsal-ventral polarity of retinal progenitor cells.

In addition to the D-V polarity of the retina, lens formation was found to be severely perturbed, as Pax6 (Figures 6E and 6F), Chx10 (Figures 6G and 6H) and AP2α (Figures 6K and 6L) expression were not found in the lens of the Phc1 homozygous mutants.

As these phenotypes are reminiscent of the *Smad7* knockout retina, where the BMP/TGF-β signal was aberrantly upregulated,^75^ we investigated the distribution of pSmad1. In the heterozygous mutant, pSmad1 was found to be specifically distributed at the ridge of the retina and in the lens (Figure 6M; arrowheads); however, in the homozygotes, the distribution was severely perturbed (Figure 6N; red arrowheads), suggesting that the BMP signal fails to be restricted.

We further analyzed the phenotype in the e14.5 knockout retina. The overall body structure was almost the same at e14.5 in the trunk region of the homozygotes; however, the body size was slightly smaller, and microphthalmia and microcephaly were evident (Figures S9A and S9B′). The sections of the retina revealed that retinal differentiation was perturbed, with reduced expression of Pax6 (Figures S9C and S9D) and no expression of Chx10 (Figures S9E and S9F) and NFIA (Figures S9G and S9H). Perturbation of lens development was also found at e14.5, with no Pax6 (Figures S9C and S9D), NFIA (Figures S9G and S9H) or Prox1 (Figures S9I and S9J). Therefore, eye formation was severely perturbed, and lens development at e11.5 was not delayed, but was a fundamental complication of development.

As the D-V patterning of the eye region was perturbed in the *Phc1*-KO embryos, we further investigated the distribution of Gli activity in the *Phc1*^−/−^ eyes. Gli proteins are Zn-finger transcription factors that mediate the Hedgehog (Hh) signal^76^ and are important for establishing D-V patterning in the retina.^77^ We employed the *Tg* (*GBS*-GFP) transgenic mouse line, where GFP expression can reflect Gli activity in a real-time manner.^78^ While Pax6 expression was comparable in the heterozygous and homozygous mutants at e9.75, GFP expression expanded from the ventral forebrain to the retinal region (Figures S9K–S9P), and GFP remained expressed in the retina at e10.5 in the *Phc1* knockout (Figures S9Q–S9W). Therefore, the essential roles of Phc1 in the establishment of the D-V patterning of the retina were also revealed by the distribution of Hh/Gli activity.

## Discussion

### The neural inducing signals and epigenetic regulation correlate with each other

Directed differentiation is generally promoted by inductive signals that activate target gene expression. In parallel, the accessibility of the gene loci is modified by chromatin remodeling, which confers the basis for gene regulation. The integration of such instructive and permissive events is essential for the proper differentiation of cells into target cell fates and to achieve committed states.^79^^,^^80^ In the case of neural induction, anti-BMP factors and FGF play inductive roles,^17^^,^^18^^,^^20^ whereas epigenetic factors provide the permissive decision.^13^^,^^14^ In this study, we have shown that not only does the anti-BMP signal induce neural gene expression but it also maintains *Phc1* expression, thereby promoting neural induction (Figures 1 and 2).^1^ Moreover, disruption of Phc1 function causes failure to restrict the expression of non-neural genes (Figures 3 and 5) and results in uncontrolled upregulation of pluripotent and non-neural genes.

The relationship between signal molecules and epigenetic regulation has been exemplified recently, where FGF promotes chromatin accessibility at neural genes.^14^ Therefore, a mutual correlation exists between these events, where inductive signals activate the expression of epigenetic factors to provide a permissive effect that leads the cells to the desired cell fate.

### Phc1 directs undifferentiated cells toward the neural lineage

In this study, we have demonstrated that Phc1 is important for early *in vitro* neural differentiation (Figures 2 and 3). Cells deficient in Phc1 function fail to suppress pluripotent genes despite being cultured in neural differentiation medium and remain in a pluripotent or early non-neural state (Figure 3). *Phc1* expression is downregulated by BMP signaling (Figure 1). This finding is consistent with the fact that Phc1 is expressed less in the GATA6-positive primitive endoderm cells that are governed by the BMP signal.^58^^,^^81^^,^^82^

Phc1 was initially isolated as a repressor of *Hox* genes in Drosophila,^83^ and its activity is conserved in vertebrates, as the aberrant upregulation of some *Hox* genes is evident in *Phc1*-KO ES cells (Figure S3).^56^^,^^57^^,^^58^ Our transcriptome analysis in *Phc1*-KO neural progenitor cells was in line with this finding; we found upregulation of some *Hox* genes, *Hoxa1* and *c13* (Figure 3; Table S2). It should be highlighted that the neural differentiation protocols employed in this study primarily yield forebrain-type neural cells; hence, the *Hox* genes, which provide positional information at the trunk level, should be quiescent. Moreover, we assume that this aberrant Hox gene expression does not facilitate posterior differentiation because the attempt to differentiate into posterior neural identities was not successful (Figure S5).

The upregulated *Cdkn2a* expression in *Phc1*-KO cells is also consistent with previous observations.^56^ Moreover, in this study, we emphasized that *Phc1*-KO cells fail to exit the stem cell state; however, some genes, including *Lhx6/9*, *Islet2* (*Isl2*), *Mag* (*Myelin Associated Glycoprotein*) and *Gata2*/*3*,^84^ which are expressed in more mature neural and neuronal cells, were found to be aberrantly upregulated. Furthermore, the genes with increased expression also include *Gata2,* which is expressed during haemopoietic differentiation,^85^ and *Krt17* and *42*^86^ in the non-neural ectoderm. Therefore, although the aberrant upregulation of pluripotent genes is evident, the overall regulation of gene expression is perturbed and causes random gene expression in *Phc1*-KO cells.

It has been widely known that Phc1 forms PRC1 together with other proteins of Ring1A/B, Cbx and Pcgf, and these subunits constantly interchange with their own paralogues during differentiation.^40^^,^^45^^,^^87^^,^^88^^,^^89^ Therefore, we propose that Phc1 acts as a modifier for complex formation. In the pluripotent state, the PRC1 composition includes Pcgf6 and Cbx7, but once neural lineage differentiation starts, these proteins are replaced with Pcgf4 and Cbx8,^40^^,^^90^ and the expression of pluripotent and non-neural genes is inhibited. Thus, in this hypothetical model, Phc1 is involved in the modification of PRC1 composition and recruits any of these co-factor proteins to provide permissive regulation on the gene expression.^40^ It is also assumable that those PRC1 proteins that are necessary for maintaining the pluripotent state are replaced with Phc1, thereby allowing neural differentiation. Our data suggest that Phc1 is dispensable for mesoderm and endoderm differentiation (Figure 4). Thus, the specificity of PRC1 target genes depends on the combinations of the PRC1 proteins.

To locate the loci affected by Phc1, we surveyed the consensus sequences of ATAC-seq peaks that were more accessible in wild-type and *Phc1*-KO cells (Figures 5B and 5C). The results show that the sequence including TAATTA,^91^^,^^92^ which can be targeted by Homeobox proteins, was most enriched in the wild-type accessible peaks (Figure S7B), and this observation is consistent with the fact that neural differentiation is promoted by a number of Homeobox proteins. Conversely, the accessible peaks in *Phc1*-KO contain the consensus sequence GGGTGTGG, one of the KLF-binding motifs (Figure S7A),^93^^,^^94^ suggesting that some pluripotent factors, including Klf4, are still accessible to the target loci even after differentiation starts. Thus, one assumable model for the Phc1 function is that it binds to some of the regions that are accessible in the *Phc1*-KO and condenses them.

The above insights raise several possibilities regarding the direct mechanisms by which Phc1 functions. As Phc1 participates in the cPRC1 complex and acts as a part of the complex, it can be speculated that Phc1/cPRC1 binds to the target DNA sequences of pluripotent and non-neural genes directly and acts to compact chromatin to make gene expression quiescent. As with other possible mechanisms, Phc1/cPRC1 may regulate a specific gene, and that gene product indirectly regulates the accessibility of pluripotent and neural genes. It can also be considered that Phc1 encourages, rather than represses, the transcription of neural genes by changing chromatin status. In addition, as proposed in a previous report,^58^ Phc1 may interact with *cis*-regulatory transcription factors (e.g., Nanog) and modulate the transcriptional activity of such transcription factors. It is also possible that Phc1 is involved in the three-dimensional chromatin structure, as shown in a previous report.^57^

In this regard, to further survey the correlations between Phc1 and other PRC factors as well as histone modifications by H3K27me3 and H2K119ub1 (repressive marks), we compared our ATAC-seq data with publicly available Chromatin Immunoprecipitation and sequencing (ChIP-seq) data.^57^ The peaks occupied by Ezh2 and Ring1B were removed during neural differentiation; however, the peak distribution was not entirely complementary to our ATAC-seq at the *Nanog*, *Bmp4* and *Pax6* loci (Figure S8). H3K27me3 and H2K119ub1 were evidently present in the *Pax6* locus in the ES cells, but they were removed during differentiation; these sites did not correspond completely to the accessible sites according to the ATAC-seq (Figure S8). Therefore, Phc1 does not seem to directly correlate with these co-factors and histone modification. Future analysis is warranted to search for the direct binding sites of Phc1.

Regarding the dynamic changes in chromatin accessibility during neural differentiation, it is notable that the forced expression of Pax6 allowed further neural differentiation even in the absence of Phc1 (Figures 5N–5U). Therefore, it would also be of interest to analyze the chromatin structure of Pax6-inducible cells.

Among the PRC1 component proteins, it has been shown that the core protein Ring1B is essential for the maintenance of stem cells and for neuronal fate specification during brain development (Figures S1A–S1D).^95^^,^^96^ On the other hand, some of the co-factor proteins are redundant on their own, particularly for the maintenance of pluripotency.^59^ A recent study demonstrated that ES cells with triple knockout of *Phc1*/*2*/*3* genes have the ability to maintain in an undifferentiated state, which supports our observation that *Phc1*-KO is dispensable for ES cell self-renewal (Figure S3).^59^ However, another report claimed that the loss of Phc1 function caused spontaneous differentiation and concluded that Phc1 is crucial for the maintenance of pluripotency.^58^ They reported that Phc1 directly binds to Nanog and potentiates its activity. We have yet to have a conclusive explanation for this discrepancy between our findings and those data.

Regardless of Phc1 roles in the maintenance of pluripotency or differentiation, the dissimilarity in phenotypes between the *in vitro* stem cell differentiation system and the *in vivo* model remains to be discussed. (Figure 6; as discussed below).^66^ In this regard, the analyses of tissue samples taken from mutant homozygotes will be informative to uncover the impact of the gene(s) *in vivo* (e.g., expression profiling in embryos).

### Phc1 is important in the late stages of embryonic development

At the mouse embryonic level, Phc1 is not involved in early neurogenesis but rather plays an essential role in organ formation at late developmental and postnatal stages. Individuals with mutations in the *PHC1* gene are described as having primary microcephaly or cerebral malformations that cause intellectual disabilities.^97^^,^^98^^,^^99^ Consistently, the *Phc1* homozygotes displayed smaller brain sizes (Figures S9A and S9B). Moreover, defects in eye-field formation during embryonic development are evident^66^ (Figure 6), which are similar to those found in mutants of other PRC factors, such as *Rybp*.^100^ *Phc1* mutant embryos also exhibit alterations in the anterior-posterior positioning of the vertebra due to *Hox* gene perturbation^56^ and perinatal lethality due to cardiac defects.^66^ Although the mechanisms behind this wide range of phenotypes remain elusive, it is clear that there are distinct mechanisms mediating the *in vivo* and *in vitro* differentiation processes.

Similar discrepancies have been observed in other gene mutants. For instance, *Pcgf6*-KO mouse ES cells undergo spontaneous differentiation and are suggested to have functions at early embryonic stages,^101^ but their individual homozygotic mice are still viable with a defective phenotype in germ cells at the adult stage.^102^ Furthermore, *Pcgf6*-KO human ES cells have impaired neuroectodermal differentiation,^103^ which is opposite to the phenotype found in mouse ES cells.

We found that the BMP4 signal is aberrantly activated in the eye-field region, which affects the D-V polarity of the neural retina and its development (Figure 6).^104^ This is one of the possible common mechanisms between the *in vitro* and *in vivo* differentiation processes (Figures 5L, 5M, 6M, and 6N). The D-V polarity of the neural retina is partly regulated by signaling molecules from the lens.^105^^,^^106^ In addition, a previous report documented the perturbation of the D-V polarity of brain development in *Ring1B* homozygotic mutants.^96^ Therefore, Gli activity was perturbed in the developing ventral retina of *Phc1*-KO mice (Figure S9). These observations suggest that partially common mechanisms exist *in vivo* and *in vitro* and that there is a correlation between Phc1 and Ring1B.

In this study, we focused on epigenetic regulation in early neural differentiation. Future studies are warranted to explore the epigenetic regulation in other stages of differentiation (e.g., neural specification and neuronal differentiation) or other lineages (e.g., mesoderm and endoderm differentiation), as different molecules might be involved. We envisage that unveiling the relationships between inductive signals and epigenetic regulation will lead to a better understanding of cell fate decisions and specification.

### Limitations of the study

As all mechanistic analyses were performed in the ES cell experimental system, the direct involvement of Phc1 during embryonic development is still elusive. Analyses of the embryonic specimens with high resolution (e.g., single-cell expression profiling and chromatin accessibility assay), especially those extracted from the retinal and lens regions, will hopefully account for the seeming phenotypic discrepancies between the two experimental systems.

## STAR★Methods

### Key resources table

**Table:** 

REAGENT or RESOURCE	SOURCE	IDENTIFIER
**Antibodies**		

Rabbit polyclonal anti-Pax6 (1:500)	Millipore	Cat#AB2237; RRID:AB_1587367
Mouse monoclonal anti-Pax6 (1:50)	Developmental Studies Hybridoma Bank	Cat#pax6; RRID: AB_528427
Rabbit polyclonal anti-Rax (1:250)	TaKaRa	Cat#M228
Rabbit polyclonal anti-Phc1/Rae28 (1:250)	Manabu Shirai; Takihara et al., 1997^66^	N/A
Sheep polyclonal anti-Green Fluorescent Protein (GFP) (1:1000)	Bio-Rad	Cat#4745-1051; RRID:AB_619712
Goat polyclonal anti-Sox1 (1:1000)	R&D Systems	Cat#AF3369; RRID:AB_2239879
Mouse monoclonal anti-Nanog (1:1000)	BD Biosciences	Cat#560259; RRID:AB_1645261
Mouse monoclonal anti-Nestin (1:500)	Abcam	Cat#ab11306; RRID:AB_1640723
Rabbit monoclonal anti-Nkx2.1 (1:1000)	Abcam	Cat#ab76013; RRID:AB_1310784
Rabbit polyclonal anti-phospho-Histone H3 (Ser10) (1:500)	Millipore	Cat#06-570; RRID:AB_310177
Mouse monoclonal anti-Gata4 (1:250)	Santa cruz	Cat#sc-25310; RRID:AB_627667
Mouse monoclonal anti-AP2α (1:250)	Santa cruz	Cat#sc-12726; RRID:AB_667767
Sheep polyclonal anti-Chx10 (1:500)	Abcam	Cat#ab16141; RRID:AB_302278
Rabbit polyclonal anti-NFIA (1:500)	Abcam	Cat#ab228897; RRID:AB_2923081
Goat polyclonal anti-Prox1 (1:500)	R&D Systems	Cat#AF2727; RRID:AB_2170716
Mouse monoclonal anti-Tbx5 (1:250)	Santa cruz	Cat#sc-515536
Rabbit monoclonal anti-phospho-Smad1 (1:500)	Cell Signaling Technology	Cat#9516; RRID:AB_491015
Rabbit polyclonal anti-HA (1:1000)	Abcam	Cat#ab9110; RRID:AB_307019
Mouse monoclonal anti-Phc1 (1:1000)	Active motif	Cat#39723; RRID:AB_2713961
Rabbit polyclonal anti-LaminB1 (1:1000)	MBL Life Science	Cat#PM064; RRID:AB_10693917
4′,6-diamidino-2-phenylindole (DAPI) (1:500)	TCI chemicals	Cat#A2412
Cy3 AffiniPure F(ab')₂ Fragment Donkey Anti-Rabbit IgG (H+L) (1:500)	Jackson ImmunoResearch	Cat#711-166-152; RRID:AB_2313568
Cy3 AffiniPure F(ab')₂ Fragment Donkey Anti-Mouse IgG (H+L) (1:500)	Jackson ImmunoResearch	Cat#715-166-151; RRID:AB_2340817
Cy3 AffiniPure F(ab')₂ Fragment Donkey Anti-Sheep IgG (H+L) (1:500)	Jackson ImmunoResearch	Cat#713-166-147; RRID:AB_2340729
Cy5 AffiniPure F(ab')₂ Fragment Donkey Anti-Mouse IgG (H+L) (1:500)	Jackson ImmunoResearch	Cat#715-606-150; RRID:AB_2340865
Fluorescein (FITC) AffiniPure F(ab')₂ Fragment Donkey Anti-Sheep IgG (H+L) (1:500)	Jackson ImmunoResearch	Cat#713-096-147; RRID:AB_2340720
Anti-rabbit IgG, HRP-linked (1:5000)	Cell Signaling Technology	Cat#7074; RRID:AB_2099233
Anti-mouse IgG, HRP-linked (1:5000)	Cell Signaling Technology	Cat#7076; RRID:AB_330924

**Chemicals, peptides, and recombinant proteins**		

Gelatin	Sigma	Cat#G-2500
poly-D-lysine	Sigma	Cat#P6407
Knockout DMEM/F-12	Thermo	Cat#12660012
Neurobasal medium	Thermo	Cat#21103049
PSG	Wako	Cat#161-23201
B27	Thermo	Cat#17504044
N2	Thermo	Cat#17502048
ChIR99021	Sigma	Cat#SML1046
PD0325901	Wako	Cat#162-25291
Monothioglycerol	Wako	Cat#195-15791
LIF	Wako	Cat#129-05601
Blasticidin S Hydrochloride	Wako	Cat#026-18701
GMEM	Thermo	Cat#11710035
KSR	Thermo	Cat#10828010
FBS	Thermo	Cat#10439001
NEAA	Thermo	Cat#11140050
Sodium pyruvate	Wako	Cat#190-14881
IMDM	Thermo	Cat#31980030
Ham’s F-12	Thermo	Cat#31765035
chemically defined lipid concentrate	Thermo	Cat#11905031
BSA	Sigma	Cat#A9418
apo-Transferrin bovine	Sigma	Cat#T1428
Matrigel	Corning	Cat#354234
SAG	Selleck	Cat#S7779
BMP4	R&D Systems	Cat#5020-BP-010
LDN193189	Selleck	Cat#S2618
DMEM/F-12/GlutaMAX	Thermo	Cat#10565018
bFGF	Thermo	Cat#PMG0031
RA	Sigma	Cat#R2625
Nodal	R&D Systems	Cat#3218-ND-025
Lipofectamine 3000	Thermo	Cat#L3000001
Puromycin	Wako	Cat#166-23153
Proteinase K	Wako	Cat#161-28701
EdU	Wako	Cat#052-08843

**Critical commercial assays**		

PicoPure RNA isolation kit	Thermo	Cat#KIT0204
NucleoSpin RNA purification kit	MACHEREY-NAGEL	Cat#U0955C
Click-iT EdU Alexa Flour 555 Imaging Kit	Invitrogen	Cat#C10338
TruSeq stranded-mRNA library preparation kit	Illumina	Cat#20020594
MinElute reaction cleanup kit	QIAGEN	Cat#28206
ThruPLEX DNA-seq kit	TaKaRa	Cat#RB4674
Amersham ECL Western Blotting Detection Reagent	Cytiva	Cat#RPN2236

**Deposited data**		

Raw RNA-seq	This paper	DDBJ: DRA016282
Raw ATAC-seq	This paper	DDBJ: DRA016281

**Experimental models: Cell lines**		

EB5 *Rax*-GFP	RIKEN BioResource Center	Cat#AES0145 (clone #20-10); RRID:CVCL_J650
EB5 *Rax*-GFP *Phc1*-KO	This paper	N/A
EB5 *Rax*-GFP *Ring1B*-KO	This paper	N/A
EB5 *Rax*-GFP *Cbx2*-KO	This paper	N/A
EB5 *Rax*-GFP *Phc1*-KO^*Phc1*-KI^	This paper	N/A
EB5 *Rax*-GFP *Phc1*-KO^*Phc2*-KI^	This paper	N/A
EB5 *Rax*-GFP *Phc1*-KO^*Tg*(*DOX-Pax6*)^	This paper	N/A

**Experimental models: Organisms/strains**		

C57BL/6J *Phc1*+/- and *Phc1*-/- mice	Manabu Shirai; Takihara et al., 1997^66^	N/A
*Tg* (*GBS*-*GFP*) mice	Kindly provided by Dr. James Briscoe (The Crick Institute, London, United Kingdom) Bakaskas et al., 2012^78^	N/A

**Oligonucleotides**		

Ring1B guide RNA (+) TGATGAGTATGAAGCGCATC (-) GACCCGAACTTTGATGCACT	This paper	N/A
Cbx2 guide RNA (-) GGTCAAGTGGCGCGGCTGGT	This paper	N/A
Phc1 guide RNA (-) TAGCCGTGGCACAGGCTTCT (+) TATGCACGTGTTCGGAGGCG (+) CTGGAACGCTATCATGGAAA	This paper	N/A
*Ring1B*-KO F: CCTACCTGTCGGAAAAAACTGGTTTCTAAA R: GGATTCTGTGAGTCTCACAAACACAAATTC	This paper	N/A
*Cbx2*-KO F: CTCTGCGGGGCTAACCGCCCGCTCTTATCT 1R: CTCGCTCCCCGTGGGCTCGTAAACAAAGGG	This paper	N/A
*Phc1*-KO F: GAACTTGGCAGTGAGGAACCAACAGGCTTC R: GCTCACAGTCACTGTTTGAGCTGCAGGTAA 2R: CGCTTGCCCTGGATCTTAGCACGAGCAATA	This paper	N/A
*Phc1*-KI F: TTAATTAGGAGCTTGGCGAGCAGC R: CTGCACTGCTTGCCGTTCATACAG	This paper	N/A
*Phc2*-KI F: TTAATTAGGAGCTTGGCGAGCAGC R: TATTGGTCCCACTGGTGTTG	This paper	N/A

**Recombinant DNA**		

*pX459* (pSpCas9(BB)-2A-Puro V2.0)	Addgene	Cat#62988; RRID:Addgene_62988
*pBluescript*-SK	Promega	N/A
*PB-TAG-ERP2*	Addgene	Cat#80479; RRID:Addgene_80479
*pCMV*-hyPBase	Yusa et al., 2009^107^	N/A

**Software and algorithms**		

CLC Genomic Workbench	QIAGEN	RRID:SCR_011853
DAVID	Huang et al., 2009^108^	RRID:SCR_001881
CRISPR-direct	Naito et al., 2015^109^	https://crispr.dbcls.jp
fastp	Chen et al., 2018^110^	RRID:SCR_016962
Bowtie2	Langmead and Salzberg, 2012^111^	RRID:SCR_016368
SAM Tools	Li et al., 2009^112^	RRID:SCR_002105
Picard tools	Broad Institute	RRID:SCR_006525
bedtools	Quinlan and Hall, 2010^113^	RRID:SCR_006646
MACS3	Zhang et al., 2008^114^	RRID:SCR_013291
featureCounts	Liao et al., 2014^115^	RRID:SCR_012919
DESeq2	Meisterernst et al., 1988^116^	RRID:SCR_015687
HOMER	Heinz et al., 2010^117^	RRID:SCR_010881
ImageJ	Schneider et al., 2012^118^	https://imagej.nih.gov/ij/; RRID:SCR_003070
Adobe Photoshop 2023	Adobe	RRID:SCR_014199
Adobe Illustrator 2023	Adobe	RRID:SCR_010279
R software (version 4.2.1)	Dessau and Pipper, 2008^119^	RRID:SCR_001905
GraphPad Prism 9 (version 9.4.1)	GraphPad	RRID:SCR_002798

**Others**		

Emerald Amp Max PCR Master Mix	TaKaRa	Cat#RR320
PrimeScript RT Master Mix	TaKaRa	Cat#RR036A
Universal qPCR Master Mix	BioLabs	Cat#M3003S

### Resource availability

#### Lead contact

Further information and requests for resources and reagents should be directed to and will be fulfilled by the lead contact, Noriaki Sasai (noriakisasai@bs.naist.jp).

#### Materials availability

ES cell lines generated in this study are available upon request from the lead contact.

### Experimental model and study participant details

#### Ethical statement

All animal experiments were performed under the approval of the Animal Welfare and Ethical Review Panel of Nara Institute of Science and Technology (approval numbers of 1810 and 2311) with the protocols in accordance with the national and internal regulations.

#### The *Phc1*-deficient mice and *Tg* (*GBS*-*GFP*)

The *Phc1*-KO mice were established previously, in which the exon 4 site had been replaced with the neomycin-resistant gene.^66^ The transgenic *Tg* (*GBS*-*GFP*) mice, where the GFP expression is driven by the Gli-binding sites, can identify the cells with active Hedgehog signal, were generously provided by Dr. James Briscoe.^78^ Breeding pairs were set up between *Tg* (*GBS*-*GFP*) and *Phc1* heterozygous mice to obtain the transgenic *Phc1* homozygous embryos for analysis. The embryos used were solely based on their genotypes, not their gender.

#### Maintenance of mouse embryonic stem cells

The mouse ES cell line *Rax*-GFP (an EB5 derivative having the knock-in *GFP* gene in the *Oct3/4* locus)^49^^,^^120^^,^^121^ was distributed by RIKEN BioResource Center (Cell Number AES0145). For the maintenance of the pluripotent state of ES cells, 7.5 x 10^4^ cells were plated onto a 35 mm culture dish (Corning; 430165) coated with 0.1 mg/ml poly-D-lysine (Sigma), and the medium was changed every other day. The maintenance medium used contains double inhibitors for Mitogen Signal-Regulated Kinase (MEK) and GSK-3β (two inhibitors, or 2i).^22^ This medium contained 1:1 of Knockout Dulbecco's Modified Eagle Medium (DMEM)/F-12 nutrient mixture (Thermo) and Neurobasal medium (Thermo), supplemented with a mixture of 1x Penicillin-Streptomycin-L-Glutamine Solution (PSG; Wako), 1x B27 (Thermo) and 1x N2 (Thermo) supplements, 6 μM of ChIR99021 (GSK-3β inhibitor; Sigma), 1 μM of PD0325901 (MEK inhibitor; Wako), 1x Leukemia Inhibitory Factor (LIF; Wako), 450 μM monothioglycerol (Wako) and 20 μg/ml of Blasticidin S Hydrochloride (Wako). At the passage immediately before the neural differentiation, the cells were cultured in culture dish coated with 0.1% gelatin (Sigma) in the GMEM-based maintenance medium^15^^,^^16^ containing Glasgow Minimum Essential Medium (GMEM; Thermo) with 10% Knockout Serum Replacement (KSR; Thermo) and 1% Fetal Bovine Serum (FBS; Thermo), supplemented with 1x Non-Essential Amino Acids (NEAA; Thermo) and 1x Sodium pyruvate (Wako), 1x LIF, 450 μM of monothioglycerol and 20 μg/ml of Blasticidin S Hydrochloride.

### Method details

#### Three-dimensional organoid differentiation assays

The three-dimensional neural differentiation was carried out using gfCDM with slight modifications.^49^ The gfCDM contains 1:1 of Iscove’s modified Dulbecco’s medium (IMDM; Thermo) and Ham’s F-12 Nutrient Mixture (Thermo), supplemented with chemically defined lipid concentrate (Thermo), 1x PSG, Bovine Serum Albumin (BSA; Sigma) and 15 μg/ml apo-Tansferrin bovine (Sigma). ES cells were seeded at a density of 3,000 - 4,000 cells/well into non-adherent 96-well U bottom culture plate (PrimeSurface 96U; Sumitomo Bakelite), and the day when the cells were seeded was defined as day 0. At day 1, growth factor-reduced Matrigel (Corning) was added to the final concentration of 2% to promote neuroepithelial structure formation and for the better cell survival. In this protocol, the diencephalic area, including the prethalamic and thalamic areas, was generated unless the differentiation was modified by the addition of exogenous signals.^51^ At the same concentration of 0.5 μM, ChIR99021 (Sigma) was treated from day 4 onward to form the retinal structure^55^ and SAG (Selleck) at day 3 for the hypothalamus,^51^ respectively. BMP4 (Figure 1; R&D Systems) and LDN193189 (Figures 5H–5M; Selleck) were used when necessary at 10 ng/ml and 50 nM, respectively, at the start of differentiation, and the cells were collected for analysis at day 4.

For embryoid bodies differentiation, the cells were cultured in GMEM-based maintenance medium, without the supplement of LIF and blasticidin, for 3 days.

For mesoderm and endoderm differentiation, the protocols were modified as previously described.^69^^,^^71^ The cells were cultured in the N2B27 medium contains 1:1 of DMEM/F-12/GlutaMAX supplement (Thermo) and Neurobasal medium, with mixture of 1x PSG, BSA, 1x N2 and 1x B27 supplements. The ES cells were cultured in N2B27 medium containing 5 ng/ml of fibroblast growth factor (bFGF; Thermo) for first two days, and subsequently added 50 nM of all-trans retinoic acid (RA; Sigma) for mesoderm differentiation and 100 ng/ml of Nodal (R&D Systems) for endoderm differentiation. The cells were cultured to day 7 for mesoderm and day 5 for endoderm differentiation.

Posterior neural differentiation was performed with a modified protocol as previously established,^67^ using the N2B27 medium. The ES cells were cultured in N2B27 medium containing 50 ng/ml of bFGF for the first two days, followed by the addition of 3 μM ChIR99021 on day 2. From day 3 onwards, the medium was changed to N2B27 medium containing 500 nM all-trans retinoic acid (RA; Sigma; R2625) or 500 nM RA and 50 nM SAG.

#### Generation of mutant ES cell lines

Mutant ES cell lines for *Ring1B*, *Cbx2* (Figure S1) and *Phc1* (Figures 2, 3, 4, and 5) were generated using the CRISPR/Cas9 mutagenesis. The guide RNAs were designed with the CRISPR-direct website (https://crispr.dbcls.jp) and the sequences used in this study are shown in the key resources table. Each guide RNA fragment was subcloned into the *pX459* (pSpCas9(BB)-2A-Puro V2.0) vector at the BbsI sites.

In this reverse transfection method, trypsinised and dissociated ES cells (2.25 x 10^5^ cells) were mixed with Lipofectamine 3000 (Thermo), and selected with 1 μg/ml of puromycin (Wako) on the next day for two continuous days. After puromycin selection, the cells were grown in the GMEM-based maintenance medium for several days until the colonies grew large enough to be picked up under the microscope. In each production, 24 colonies were picked up, trypsinised and replated onto a gelatin-coated 48-well culture plate to grow for several days. The genomic DNA was extracted after subsequent passaging, and the cell pellets were dissolved in genome lysis buffer containing 100 mM Tris-HCl pH8.0, 5 mM EDTA pH8.0, 0.2% sodium dodecyl sulfate (SDS) and 200 mM sodium chloride, and added with 20 μg/ml Proteinase K (Wako). The genotypes were determined by PCR (Emerald Amp Max PCR Master Mix; TaKaRa). The primer sequences used for the PCR are shown in the key resources table.

For *Phc1*-KO cells, we obtained two clones #2 and #7, which were confirmed to have lost the Phc1 function. The clone #2, which had a more severe phenotype, was mainly analysed in this study.

#### Generation of knock-in (KI) Phc1 and Phc2 cells

The full coding sequences of Phc1 and Phc2 were modified to carry the N-terminal hemagglutinin (HA) tag sequence, and the homology arms of the upstream 0.8kb and downstream 1.1kb around the start codon of the *Phc1* gene locus (Figure S2C). Both targeting constructs were subcloned into the *pBluescript*-SK vector (Promega) and co-transfected with *pX459*, which conveys the guide RNA sequence, into the *Phc1*-KO#2 cells. The genotypes of selected KI cells were confirmed with PCR.

For generating the *Phc1*-KO^*Tg*(*DOX-Pax6*)^ cells, the modified *PB-TAG-ERP2* vector (Addgene #80479) that conveys the *Pax6* gene was transfected with *pCMV*-hyPBase^107^ into the ES cells and the transfected cells were selected with 1 μg/ml of puromycin. Three clones were picked up, and the insertion was checked by genomic PCR. For induction of the gene, 100 ng/ml of doxycycline was applied on day 3.

#### Reverse transcription and quantitative PCR (RT-qPCR)

Total RNA was extracted with the PicoPure RNA isolation kit (Thermo) or NucleoSpin RNA purification kit (MACHEREY-NAGEL) and was quantified with NanoPhotometer (IMPLEN). 500 ng of RNA was converted into complementary DNA (cDNA) with PrimeScript RT Master Mix (TaKaRa). Quantitative PCR (qPCR) was performed by Luna Universal qPCR Master Mix (BioLabs) with the primer sequences listed in Table S1.

#### Immunofluorescence

Cell aggregates (organoids) or mouse embryos were fixed with 4% paraformaldehyde for 1 hr and subsequently incubated overnight with 15% (w/v) sucrose in phosphate buffer serine (PBS) at 4°C. The specimens were embedded in OCT compound (Sakura), and the sections were prepared with Tissue Polar cryostat (Sakura Finetek) at 10 to 12 μm thickness. For immunostaining, the slides were stained overnight with primary antibodies at 4°C, followed by secondary antibodies and 4’,6-diamidino-2-phenylindole (DAPI) for 2 hrs at room temperature. The stained tissues were mounted with glycerol. The antibodies used are listed in the key resources table. For EdU labelling, ES cells were cultured to 80% confluency and then incubated in medium containing 2.5 ng/μl EdU (Wako) for 2 hrs. Cells were fixed with 4% paraformaldehyde for 15 mins and labelled with the Click-iT EdU Alexa Flour 555 Imaging Kit (Invitrogen), according to the manufacturer’s instructions.

#### Western blot

10 mg of total protein extracted from the ES cells was separated by 10% polyacrylamide electrophoresis. The gels were blotted onto a PolyVinylidene DiFluoride (PVDF) membrane and incubated with the primary and secondary antibodies denoted in the key resources table. The signals were developed by ECL™ Prime Western Blotting Detection Reagent (Cytiva) in an Image Quant 4000 CCD imager (Fujifilm).

#### mRNA sequencing

Two clones of *Phc1*-KO ES cells (KO#2 and KO#7), and wild-type ES cells were differentiated for 4 days using the gfCDM/MG protocol. Each genotype has two biological replicates. The cDNA libraries were synthesised using the TruSeq stranded-mRNA library preparation kit (Illumina) and analysed on the NextSeq 500 (Illumina), with approximately twenty million reads obtained from each genotype.

Raw FASTQ formatted sequence reads were imported into CLC Genomic Workbench (QIAGEN). Reads were then mapped to the genome obtained from the Ensembl database (GRCm38/mm10, Ensembl release 102). Next, the “Differential Expression for RNA-seq” toolset was used to calculate the fold change and significance of difference in gene expression between *Phc1*-KO and wild-type ES cells. Gene expression level was shown as reads per kilobase of exon per million mapped reads (RPKM).^122^ Pathway analyses (Figures 3C, 3D, and S4) were carried out using the Database for Annotation, Visualization and Integrated Discovery (DAVID; https://david.ncifcrf.gov).^108^

#### ATAC sequencing

To obtain the open chromatin areas, we utilised the OMNI-ATAC-seq protocol with minor modifications.^123^ The nuclei were isolated from 5x10^4^ cells of the wild-type and *Phc1*-KO differentiated for 4 days in Nuclei EZ lysis buffer (Merck), and debris was removed with 70 μm of cell strainer. The open chromatin areas were isolated by using the Tagment DNA TDE1 enzyme (Illumina) and cleaned up with the MinElute reaction cleanup kit (QIAGEN). The DNA libraries were generated using the ThruPLEX DNA-seq kit (TaKaRa), and sequenced by a NextSeq 500 (Illumina). Approximately 5x10^7^ reads were obtained from each sample.

For data analysis, the sequence reads were trimmed using fastp with default parameters^110^ and aligned to the mouse reference genome (GRCm38/mm10) using Bowtie2 with parameters; --very-sensitive -X 2000 -p 10.^111^ After filtering reads from mitochondrial DNA, we included properly paired reads through SAM Tools for further analysis.^112^ The reads duplicated by PCR were removed using the Picard tools with the Mark Duplicates program (http://broadinstitute.github.io/picard/). Reads in blacklisted regions (https://mitra.stanford.edu/kundaje/akundaje/release/blacklists/mm10-mouse/mm10.blacklist.bed.gz) were removed using bedtools.^113^ The resulting reads were used to generate the bigwig files for visualization in the genome browser using the bam coverage command with parameters; the bamCoverage command with parameters --binSize 10 --smoothLength 40 -extendReads 150 --effectiveGenomeSize 2150570000 --ignoreForNormalization chrX --normalizeUsing RPGC.

ATAC-seq peak regions of each sample were called using MACS3 with parameters -f BAMPE –nomodel -g mm --call-summits --shift -37 --extsize 74 --keep-dup all -B --SPMR -q 0.^114^ To generate a consensus set of unique peaks, we next merged ATAC-seq peaks for which the distance between the proximal ends was less than 10 base pairs by using bedtools.^113^ In total, we identified 88,459 peaks from wild-type and *Phc1*-KO samples. For each sample, the reads were counted across each peak region by using feature counts from the subread package with parameters -p -B -T 5.^115^ We calculated fold change and significance of difference in number of reads in each peak region between *Phc1*-KO ES cells and wild-type ES cells by using DESeq2 with default parameters.^116^

For the MA plots in Figure 5A, mean ATAC-seq signal between *Phc1*-KO and wild-type for each gene ('baseMean') and fold changes of ATAC-seq signal between KO and wild-type ('log2FoldChange') were calculated by DESeq2. Peaks that showed FDR-adjusted *p*-values less than 0.01 were defined as differentially accessible peaks (Figures 5B–5G). HOMER findMotifsGenome.pl was used to investigate the motif enrichment of differentially accessible peaks compared with all peaks with default parameters (Figure S7).^117^ The distance between peaks and gene regions was calculated using bedtools.^113^

### Quantification and statistical analysis

Images were captured by LSM710 and LSM980 confocal microscopes (Zeiss), and processed using Image J (National Institute of Health, USA) and Photoshop software (Adobe). Figures were prepared using Illustrator (Adobe), volcano plots and heatmap with R software (version 4.2.1) and bar graphs with GraphPad Prism (version 9.4.1). The significant differences were evaluated using a two-tailed Student’s t*-*test (for comparisons between two groups). Statistical comparisons with *p* < 0.05 were considered significant. *p*-values (∗ *p* < 0.05; ∗∗ *p* < 0.01; ∗∗∗ *p* < 0.001; ∗∗∗∗ *p* < 0.0001) are indicated in each graph.

## Data Availability

• Raw RNA-seq and ATAC-seq data are available in the DNA Data Bank of Japan (DDBJ, https://www.ddbj.nig.ac.jp/index-e.html) with accession numbers DRA016282 (RNA-seq) and DRA016281 (ATAC-seq). • This paper does not report original code. • Any additional information required to reanalyse the data reported in this paper is available from the lead contact upon request.

## Supplemental information

Document S1. Figures S1–S9

Table S1 The RT-qPCR primer sequences used in this study, related to Figures 1, 2, 3, and 4

Table S2 The list of the genes whose expression was altered in the Phc1-KO compared to those in wild-type, related to Figure 3

Table S3 The list of the genes that were found to be altered in another clone of Phc1-KO cells compared to those in wild-type, related to Figure 3

Table S4 The list of the peaks whose accessibility was altered in the Phc1-KO compared to those in wild-type, related to Figure 5

Table S5 Numerical data for bar graphs, related to Figures 1J, 2B, 3C–3E, 4C, 4F, 4I, 4M, S1D, S3A, S3D, S3G, S3J, S4B, S4C, and S5N
