# Acquisition of neural fate by combination of BMP blockade and chromatin modification

**DOI:** 10.1016/j.isci.2023.108257

**Published:** 2023-10-22

**Authors:** Agnes Lee Chen Ong, Toshiya Kokaji, Arisa Kishi, Yoshihiro Takihara, Takuma Shinozuka, Ren Shimamoto, Ayako Isotani, Manabu Shirai, Noriaki Sasai

## Main text

(iScience *26*, 107887, October 20, 2023)

In the originally published version of this article, the affiliation of Yoshihiro Takihara was incorrect. The correct affiliation of Yoshihiro Takihara is Japanese Red Cross Kinki Block Blood Center, 7-5-17 Saitoasagi, Ibaraki, Osaka 567-0085, Japan. This error has now been corrected in the article online. The authors apologize for any confusion this error may have caused.

