# Supplementary material for: Acquisition of neural fate by combination of BMP blockade and chromatin modification
Source: iScience. 2023 Sep 9;26(10):107887. doi: 10.1016/j.isci.2023.107887 (PMC10522999; doi:10.1016/j.isci.2023.107887)
Supplement: Document S1. Figures S1–S9 [file mmc1.pdf]

iScience, Volume *26*

Supplemental information

Acquisition of neural fate by combination of BMP blockade and chromatin modification

Agnes Lee Chen Ong, Toshiya Kokaji, Arisa Kishi, Yoshihiro Takihara, Takuma Shinozuka, Ren Shimamoto, Ayako Isotani, Manabu Shirai, and Noriaki Sasai

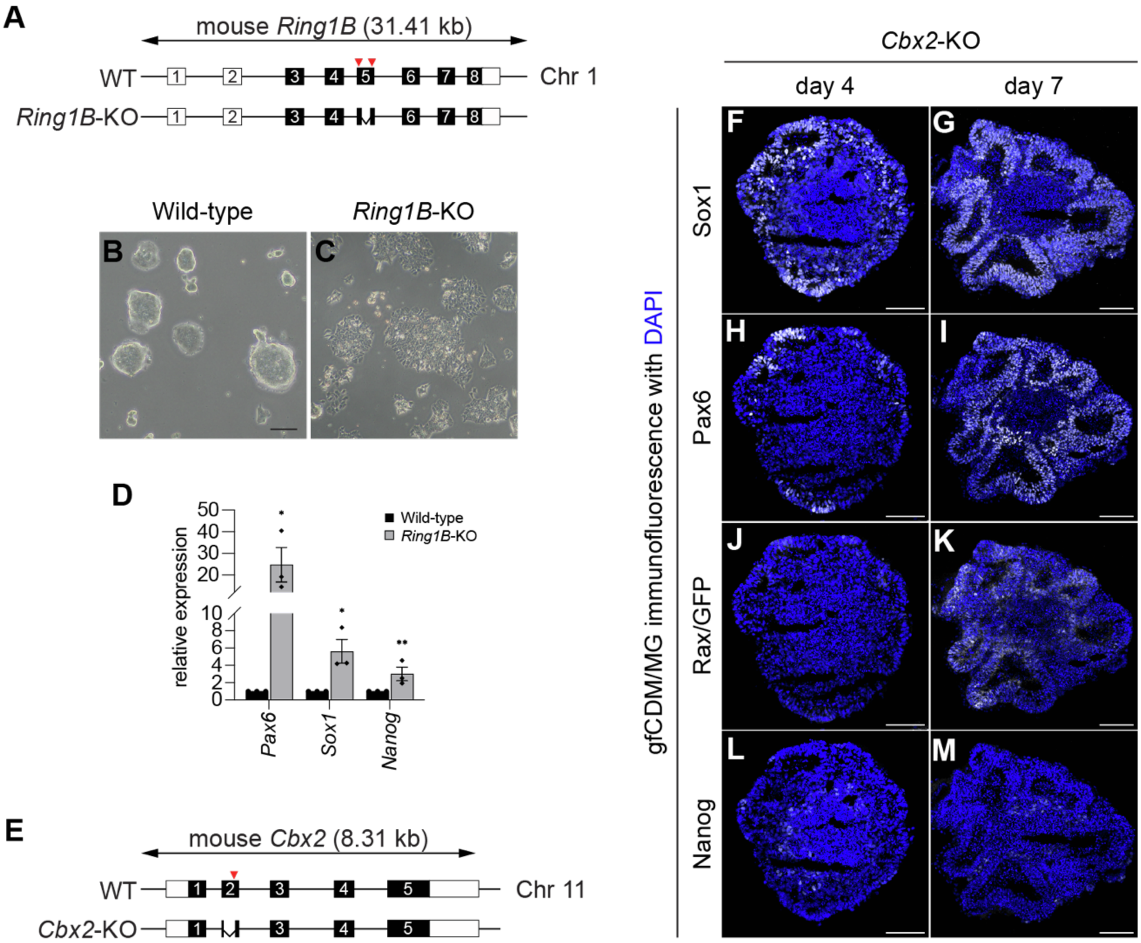

Figure S1 Generation of the mutant ES cells deficient in each of the PRC1 factors, related to Figure 2.

(A) Schematic representation for generating the *Ring1B*-KO. Two guide RNAs were designed within exon 5 of the *Ring1B* gene locus on Chromosome 1 (Chr1). (B-D) *Ring1B*-KO causes spontaneous differentiation. Gross appearance of the wild-type (B) and the *Ring1B*-KO (C) cells cultured in the maintenance medium. (D) Expression of differentiated genes on the ES cells, as analysed by RT-qPCR. Data are represented as mean ± SEM. Statistical differences were calculated using two-tailed Student's *t*-test. * indicate statistically significant $p < 0.05$ and ** indicate statistically significant $p < 0.01$. (E) Schematic representation for generating the *Cbx2*-KO. A guide RNA was designed to target the sequence in exon 2 on Chr11. (F-M) The *Cbx2*-KO cells do not have any evident phenotype for the neural differentiation. The *Cbx2*-KO cells were differentiated for four days (F,H,J,L) or for seven days with the treatment of ChIR99021 from day 4 onward (G,I,K,M), and the cells positive for Sox1 (F,G) Pax6 (H,I) and Rax/GFP (J,K) and Nanog (L,M) were analysed. Scale bars in B (for B and C) = 200 µm, (F-M) = 100 µm.

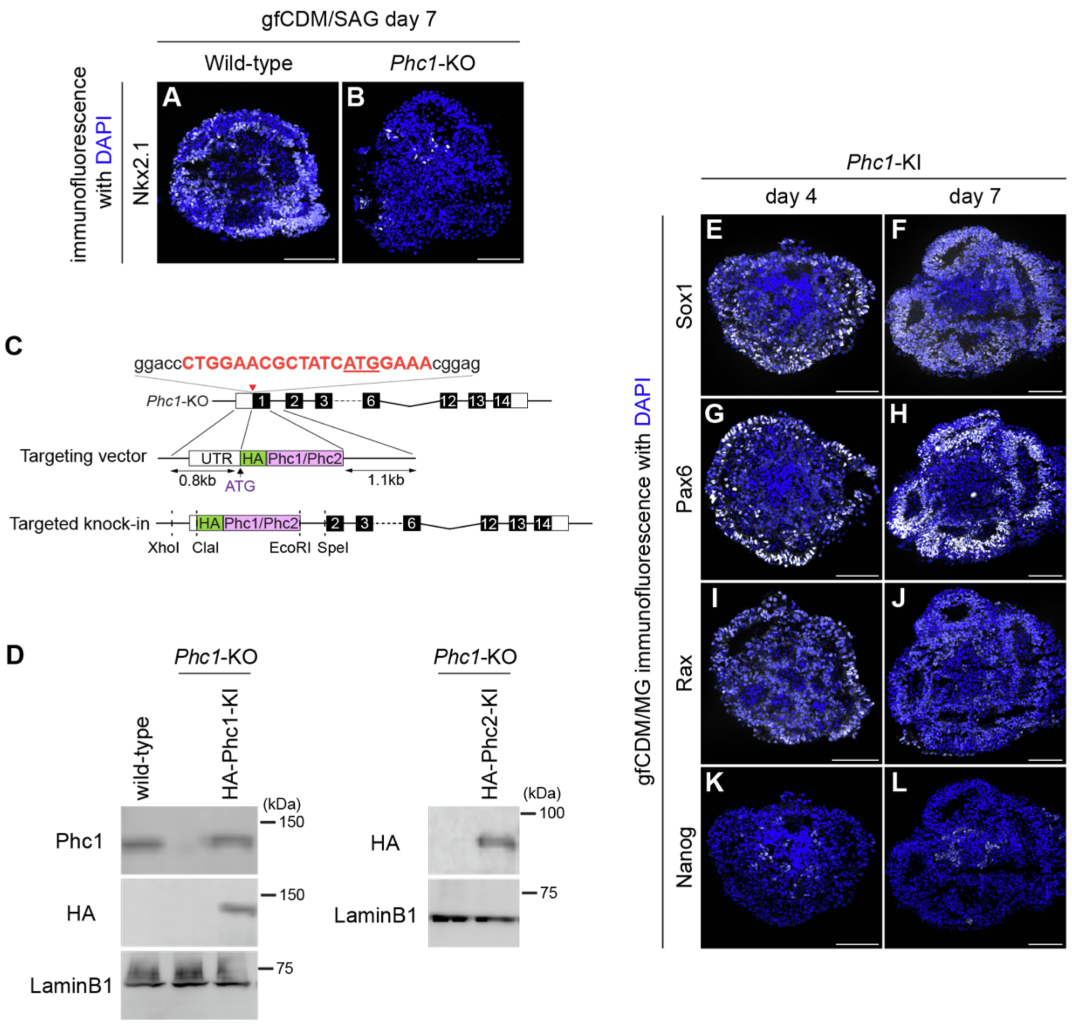

Figure S2 Phc1 is required for hypothalamic development and can be rescued by knocking-in the coding region of Phc1, related to Figure 2.

(A-B) Hypothalamic differentiation of the ES cells. Wild-type (A) or *Phc1*-KO (B) cells were differentiated for seven days with the treatment of SAG from day 3 onward for hypothalamus differentiation, and cells were analysed with the Nkx2.1 (A,B) antibody. (C) Schematic representation for generating the *Phc1*-knocked-in or Phc2-knocked-in cell line. The DNA sequence with the red capital letters indicates the targeted site of the guide RNA, and the underlined ATG sequence is the endogenous start codon. (D) Western blot analysis verifying the expression of knocked-in genes. ES cells of wild-type, *Phc1*-KI and *Phc2*-KI (used in Figure 2R-Y) cells were harvested and subjected to western blots. The anti-Phc1 antibody detected the endogenous and inserted Phc1, and the HA antibody detected the inserted Phc1 and Phc2. LaminB1 is the internal control. (E-L) The *Phc1*-KI cells can rescue the phenotype caused by the *Phc1*-KO. The *Phc1*-KI cells were differentiated for four days (E,G,I,K) or for seven days with the treatment of ChIR99021 from day 4 onward (F,H,J,L), and the cells positive for Sox1 (E,F) Pax6 (G,H) and Rax (I,J) and Nanog (K,L) were analysed. Scale bars in (A,B,E-M) = 100 μm.

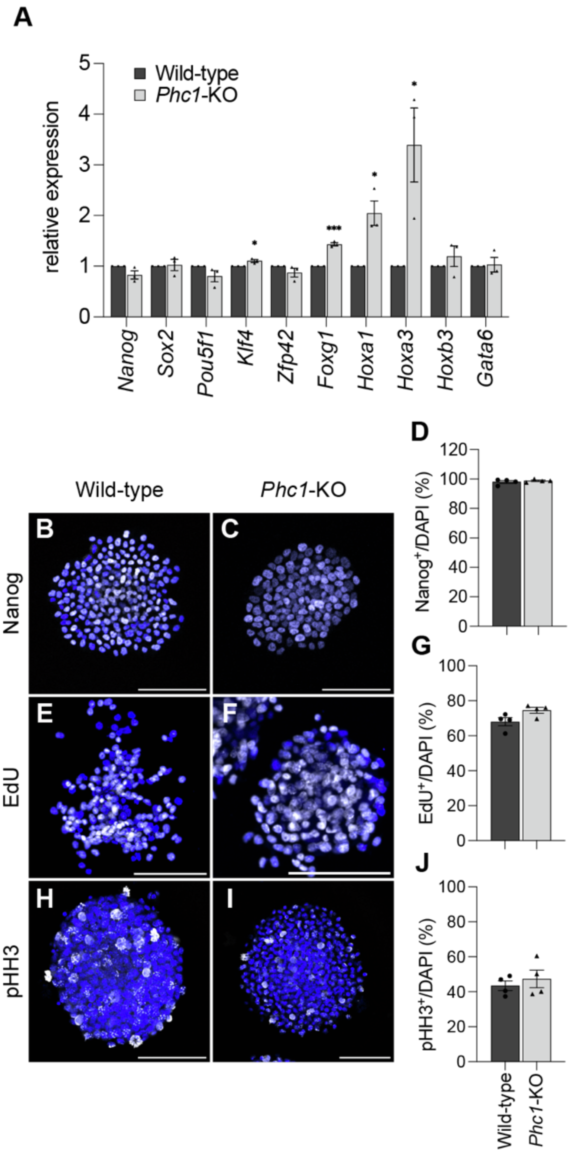

Figure S3 *Phc1* is redundant for ES cell self-renewal, related to Figure 2.

(A) RT-qPCR analysis on the wild-type and *Phc1*-KO ES cells. Note that the expression data for *Nanog*, *Klf4* and *Zfp42* are identical to those in Figure 3E. (B-I) Characterisation of the wild-type (B,E,H) and *Phc1*-KO (C,F,I) ES cells. (B-D) The immunofluorescent analysis of Nanog. (E-J) The EdU-positive cells (S-phase; E-G) and pHH3-positive cells (M phase; H-J). (D,G,J) Quantification of the positive cells over all the DAPI-positive cells. Scale bars in (B-I) = 100 μm. Data are represented as mean ± SEM. Statistical differences were calculated using two-tailed Student's *t*-test. * indicate statistically significant $p < 0.05$ and *** indicate statistically significant $p < 0.001$.

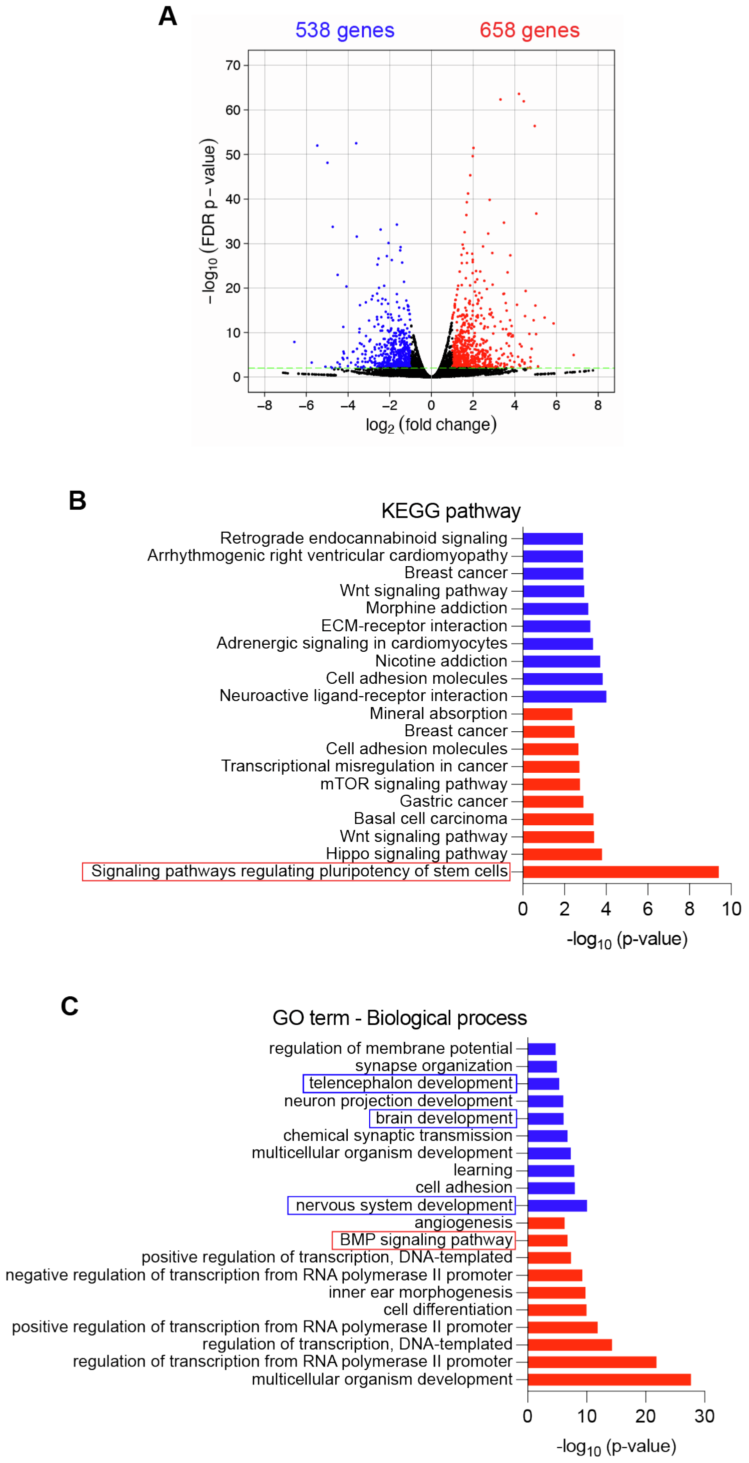

Figure S4 mRNA sequencing analysis in comparison with another *Phc1*-KO line and wild-type, related to Figure 3.

(A) Volcano plots show that the phenotypes found in the *Phc1*-KO are essentially the same as those found in another clone (Figure 3). (B,C) Enrichment analyses of KEGG pathway (B) and GO biological process (C). The detailed information (gene list) is provided in Table S3.

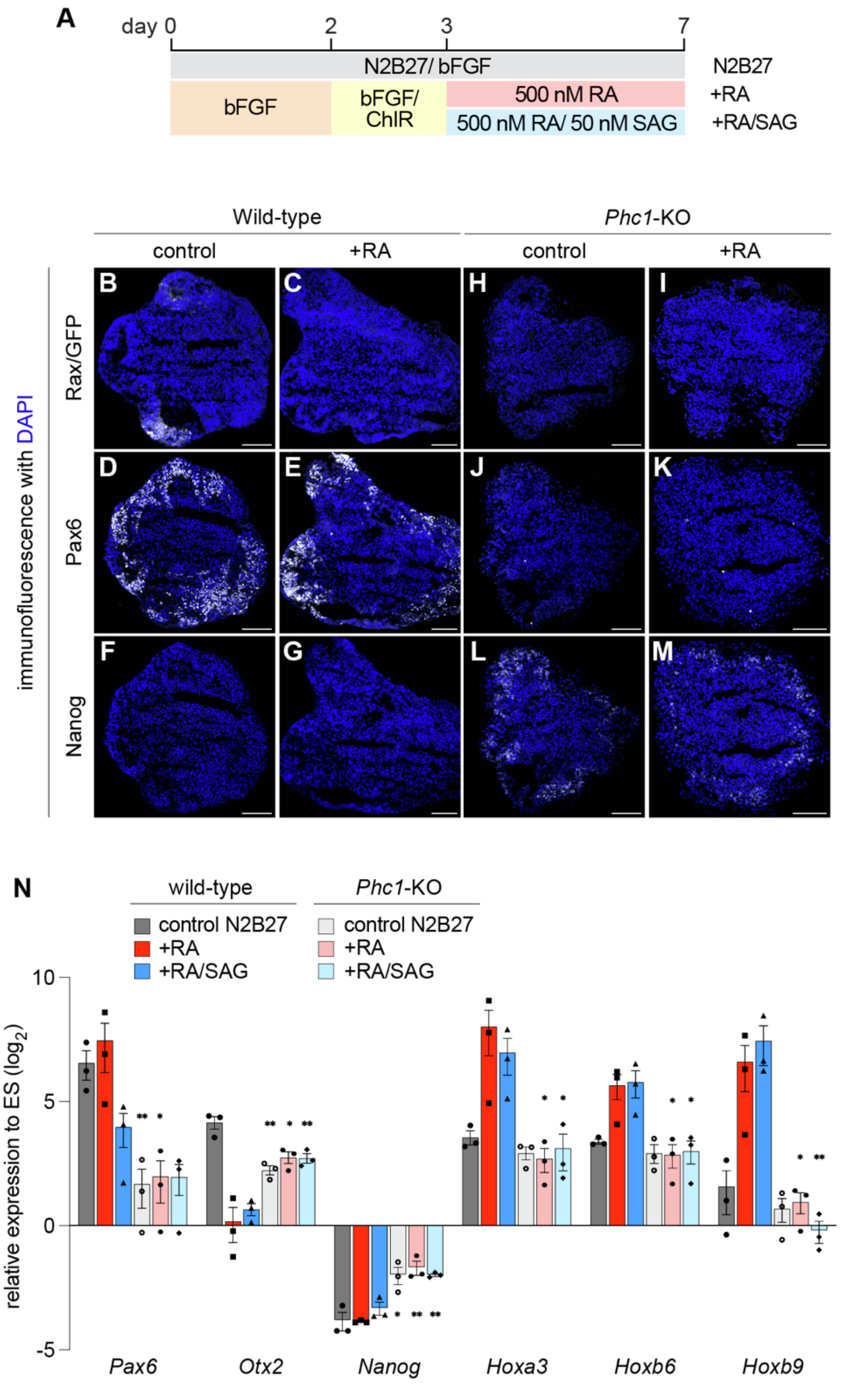

Legends on the next page

Figure S5 Posterior neural differentiation is perturbed in the *Phc1*-KO, related to Figure 3.

(A-N) Schematic representation of posterior neural differentiation for seven days (A). Wild-type (B-G) and *Phc1*-KO (H-M) cells differentiated with N2B27 + 50 ng/ml bFGF (control) and N2B27 + 3 μM ChIR99021 + 500 nM RA, or N2B27 + 3 μM ChIR99021 + 500 nM RA and 50 nM SAG. The expression of Rax/GFP (B,C,H,I), Pax6 (D,E,J,K) and Nanog (F,G,L,M) was analysed by immunofluorescence, comparing control and cells induced with RA. Scale bars = 100 μm (B-M). (N) RT-qPCR analysis of the indicated genes in wild-type and *Phc1*-KO cells at day 7. Data are represented as mean ± SEM. Statistical differences were calculated using two-tailed Student's *t*-test. * indicate statistically significant $p < 0.05$ and ** indicate statistically significant $p < 0.01$.

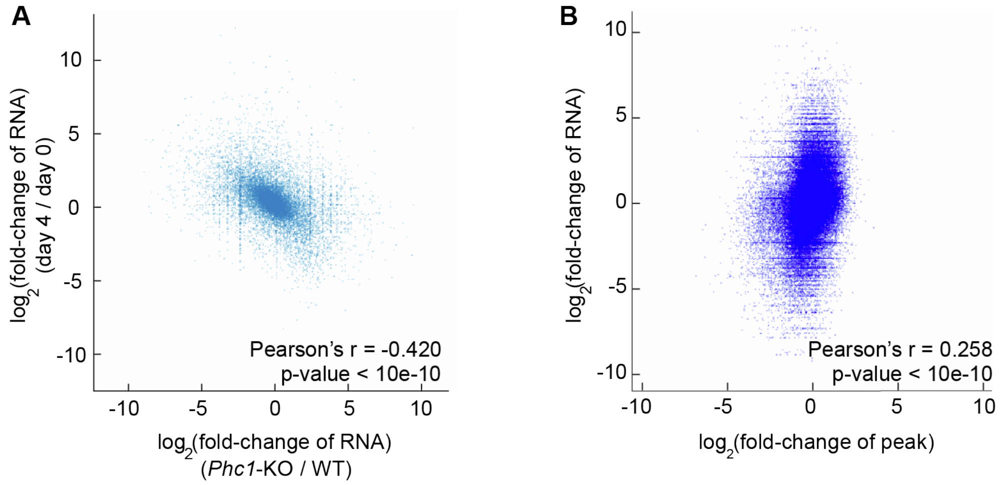

Figure S6 A comparative analysis of *Phc1*-KO cells with wild-type cells or a publicly available analysis of ES cells and neural progenitor gene expression, related to Figure 5.

(A) Fold change in gene expression of *Phc1*-KO relative to WT (wild-type; horizontal axis) was compared with that on day 4 neural cells relative to the ES cells (Bunina et al., 2020) (vertical axis).(B) The correlation between the changes of the peak signals (horizontal-axis; gained from the ATAC-seq) and the changes of the gene expression levels (vertical-axis) between the *Phc1*-KO and the wild-type (gained from the mRNA-seq). Each peak was corresponded to its closest gene, and genes whose RPKM were less than 0.1 in all samples were removed.

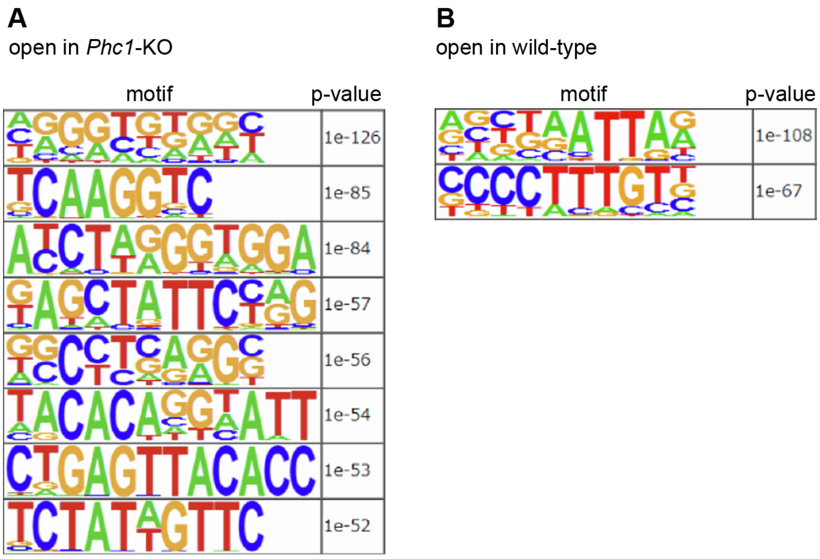

Figure S7 A motif enrichment analysis of peaks more accessible in the wild-type and those in the *Phc1*-KO, related to Figure 5.

The consensus sequences accessible in *Phc1*-KO (A) and in wild-type (B), with *p*-values lower than e-50 are shown.

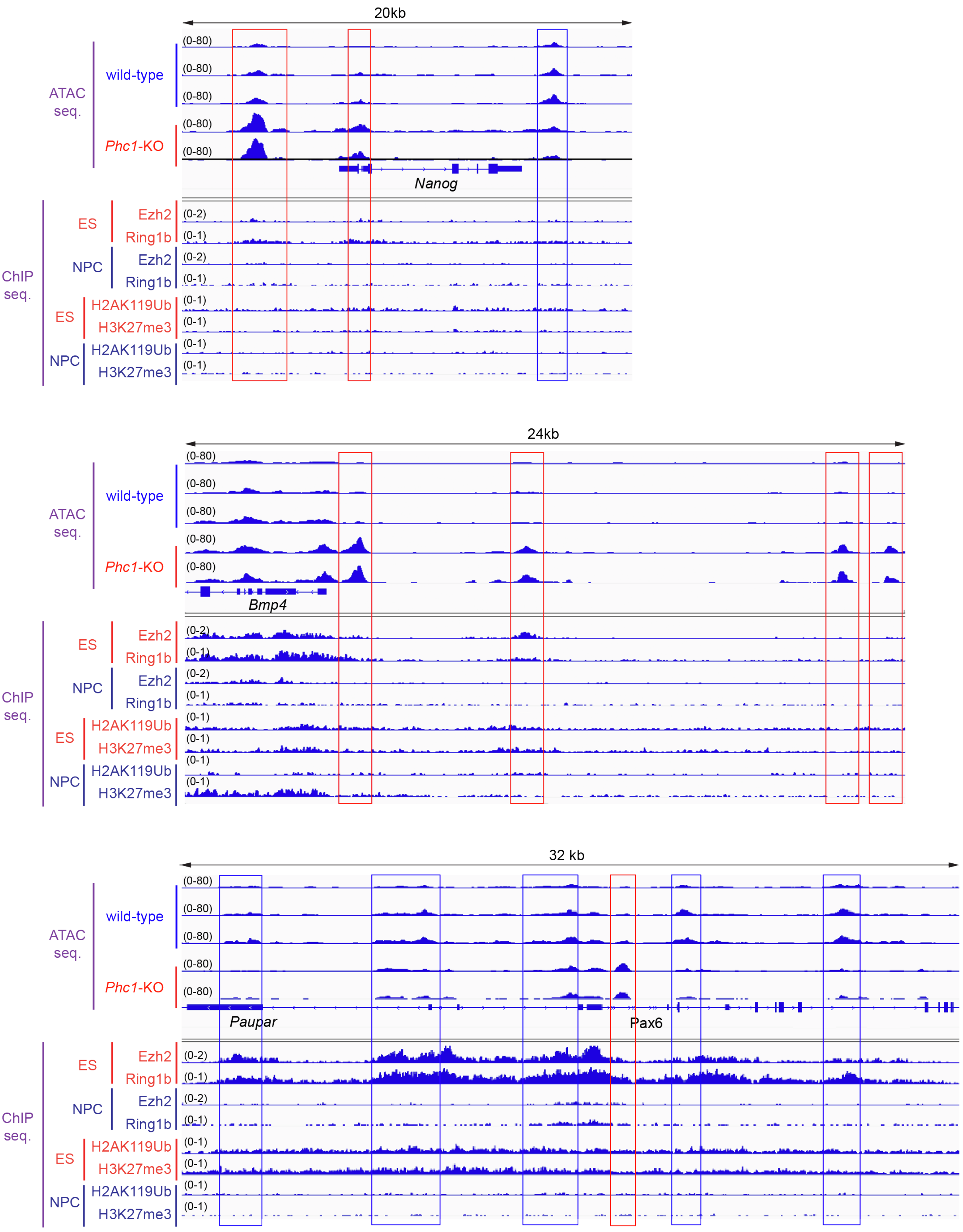

Legends on the next page

Figure S8 The comparison of the ATAC-seq peaks with ChIP-seq peaks of PRC proteins and histone modification, related to Figure 5.

The peaks obtained from the ATAC-seq analysis (Figure 5) were compared with those from the ChIP-seq analysis of ES cells and neural progenitor cells in (Kundu et al., 2017). The BigWig files of Ezh2 (SRX2353883, SRX2353889), Ring1b (SRX2353878, SRX2353885), H2AK119Ub (SRX2353882, SRX2353888) and H3K29me3 (SRX2353884, SRX2353890) were downloaded from the ChIP-Atlas database (Oki et al., 2018). The regions accessible in the wild-type and *Phc1*-KO are indicated with blue and red rectangles, respectively.

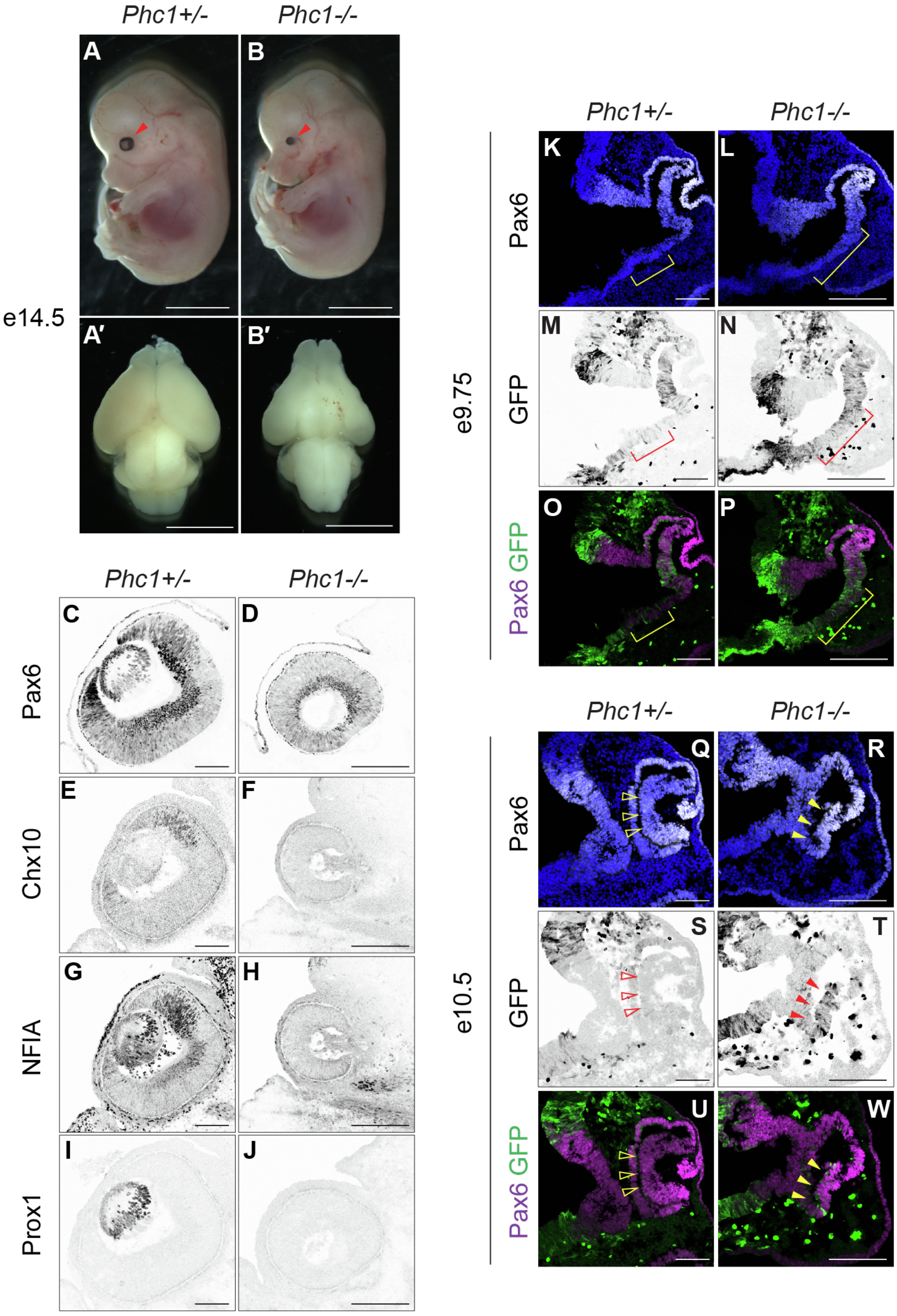

Legends on the next page

Figure S9 Additional analyses on the *Phc1-/-* embryos, related to Figure 6.

(A,B) Appearance of *Phc1*+/- (A) and *Phc1*-/- (B) and their brains (A',B') at e14.5. Evident microphthalmia (red arrowheads; A,B) and microcephaly (A',B') are recognised. Scale bars in (A,B) = 2 mm, in (A',B') = 500 μm. (C-J) Retinal cell differentiation is perturbed in the *Phc1* homozygotic mutants. *Phc1*+/- (C,E,G,I) and *Phc1*-/- (D,F,H,J) embryos were analysed by immunofluorescence with anti-Pax6 (C,D), Chx10 (E,F), NFIA (G,H) and Prox1 (I,J) antibodies. Confocal images with black/white inverted. (K-W) The distribution of Gli activity is perturbed in the *Phc1*-/- retina. The *Phc1*+/- (K,M,O,Q,S,U) and *Phc1*-/- (L,N,P,R,T,W) with *Tg(GBS-GFP)* embryos at e9.75 (K-P) and e10.5 (Q-W) were analysed with Pax6 (K,L,Q,R) and GFP (M,N,S,T) antibodies. Merged images in (O,P,U,W). (S,T) Confocal images with black/white inverted. The GFP positive areas are indicated with brackets (K-P) or with arrowheads (R,T,W), while the areas negative for GFP are indicated with open arrowheads (Q,S,U). Scale bars in (C-W) = 200 μm.

References

Bunina, D., Abazova, N., Diaz, N., Noh, K.M., Krijgsveld, J., and Zaugg, J.B. (2020). Genomic Rewiring of SOX2 Chromatin Interaction Network during Differentiation of ESCs to Postmitotic Neurons. Cell Syst *10*, 480-494 e488.

Kundu, S., Ji, F., Sunwoo, H., Jain, G., Lee, J.T., Sadreyev, R.I., Dekker, J., and Kingston, R.E. (2017). Polycomb Repressive Complex 1 Generates Discrete Compacted Domains that Change during Differentiation. Molecular cell *65*, 432-446 e435.

Oki, S., Ohta, T., Shioi, G., Hatanaka, H., Ogasawara, O., Okuda, Y., Kawaji, H., Nakaki, R., Sese, J., and Meno, C. (2018). ChIP-Atlas: a data-mining suite powered by full integration of public ChIP-seq data. EMBO Rep *19*.
